# Surface‐Engineered Filters for Wettability‐Driven Collection of Airborne Fungal Spores

**DOI:** 10.1002/gch2.70127

**Published:** 2026-07-01

**Authors:** Hafiza Umaima Affan, Claire Lenehan, Sally Fryar, Michael Taylor, Harriet Whiley, Iliana Delcheva, Melanie MacGregor

**Affiliations:** ^1^ College of Science and Engineering Flinders University Bedford Park South Australia Australia; ^2^ Flinders Institute for Nanoscale Science and Technology Flinders University Bedford Park South Australia Australia; ^3^ WSP Australia Pty Limited Adelaide South Australia Australia; ^4^ Nano and Microplastics Research Consortium Flinders University Bedford Park South Australia Australia

**Keywords:** environmental monitoring, exposure risk, fungal spore capture, indoor air quality, plasma polymerization, surface‐engineered filters

## Abstract

Environmental sampling of fungal spores is critical for assessing exposure risks, but current methods often miss low‐abundance or spatially dispersed spores, highlighting the need for more sensitive sampling methods. This study explores the use of plasma polymerization to chemically modify air filters for enhanced fungal spore capture. Polyethylene terephthalate (PET) filters are coated with nanothin films from four monomers ‐acrylic acid, 2‐methyl‐2‐oxazoline (POX), 1,7‐octadiene, and perfluorooctane (PFO)‐ and characterized using ellipsometry, X‐ray photoelectron spectroscopy, and contact angle measurements to evaluate film thickness, chemistry, and wettability. A custom aerosolization chamber was used to test the capture efficiency of plasma‐modified filters for airborne spores from four species: *Aspergillus niger*, *Cladosporium* sp., *Penicillium roqueforti*, and *Rhodotorula glutinis*. Quantitative analysis using hemocytometry and dry biomass measurement reveals species‐specific adhesion patterns that are predominantly driven by surface chemistry. Hydrophobic PFO‐coated filters achieved the highest capture of filamentous fungi, while hydrophilic POX coatings best captured the tested yeast. Coating thickness had no significant effect, highlighting the primacy of surface chemistry over film depth. These findings establish plasma polymerization as an effective strategy to tailor filter surfaces for selective fungal spore capture, providing a proof‐of‐concept for functionalized air filters that support improved bioaerosol monitoring in built environments.

## Graphical abstract

Plasma‐polymerized coatings are used to tune air filter wettability for selective airborne fungal spore capture. Hydrophobic PFO surfaces retain filamentous fungal spores, while hydrophilic POX surfaces preferentially capture *Rhodotorula glutinis*. The findings reveal that surface chemistry dominates over coating thickness and filter geometry in driving species‐specific bioaerosol collection.

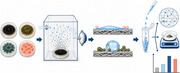

## 1 Introduction

Indoor air quality is a major factor influencing human health, as people typically spend over 80% of their time indoors [1, 2]. The major biological contaminants of indoor environments include fungi, bacteria, viruses, pollen, etc [3]. Airborne fungal spores in particular are known to exacerbate asthma, trigger allergic reactions, airway inflammation, and cause severe respiratory infections (sinusitis and bronchitis) in vulnerable individuals [4]. Globally, fungal diseases impact nearly one billion people and account for more than 1.5 million deaths annually. This already substantial burden has been further amplified by the COVID‐19 pandemic, which has led to a notable increase in fungal infections among COVID‐19 patients [5, 6]. Fungi are a diverse group of eukaryotic organisms that include yeasts, molds, and mushrooms. They form their own biological kingdom, “Kingdom Fungi,” and are distinct from plants, animals, and bacteria. These organisms that obtain nutrients by absorption have chitin in their cell walls and reproduce through spores [7]. Many species are capable of thriving in indoor environments, particularly when water intrusion, humidity fluctuations, or tightly sealed, temperature‐buffered spaces create favorable microclimates [8]. Numerous fungi release dry spores that detach easily and remain airborne for extended periods [9], enabling widespread distribution through heating, ventilation, and air‐conditioning (HVAC) systems, especially when HVAC filters are poorly maintained [10]. For example, *Aspergillus*, *Penicillium*, *Cladosporium*, and *Candida* species have commonly been found in air samples collected from various indoor environments, including homes, workplaces, hospitals, and even household furniture [2]. Given these widespread exposures, effective monitoring and control strategies are increasingly essential to manage the human health risks associated with indoor fungi. Accurate assessment of airborne fungal spores underpins indoor air quality evaluation, allergen exposure assessment, and broader environmental monitoring. Current approaches to sampling fungal spores are broadly categorized as passive or active methods, each with limitations. Passive sampling relies on gravitational settling of spores onto exposed surfaces, typically agar plates, over time. This approach is simple and inexpensive, but it provides only qualitative or semi‐quantitative data and favors larger or heavier spores. In addition, fast‐growing species, such as *Aspergillus* and *Penicillium*, may overgrow agar plates, masking slower‐growing species [11, 12, 13].

In contrast, active sampling draws a known volume of air through devices such as impaction samplers, impingers, or filter‐based systems, capturing spores onto a collection medium [14, 15]. Impaction‐based samplers, where spores impact solid agar surfaces, allow quantification and cultivation of viable spores but are limited to viable organisms [12]. Impingement, which traps spores in liquid media for downstream analysis, can compromise spore viability due to mechanical stress or osmotic effects [16, 17].

Filter‐based samplers are also commonly employed to capture airborne fungal spores by passing air through filter media like PTFE, PET, cellulose, or polycarbonate membranes, which retain the spores for subsequent analysis using microscopy or molecular methods [18]. The capture efficiency is influenced by the filter type, pore size, airflow rate, and spore characteristics (size, shape, surface properties) [19, 20]. Despite their widespread use, filter‐based samplers are generally non‐size‐selective unless paired with aerodynamic classifiers, leading to underrepresentation of small spores like *Aspergillus* (∼2–5 µm) or overloading when capturing large spores like *Alternaria* (20–60 µm). Small (<2 µm), deformable spores may bypass, bounce off or detach from filters, especially under high airflow conditions or due to vibration during sampling [19, 21]. Desiccation can also compromise the viability and morphology of spores, affecting RNA/DNA integrity and yield, while low affinity for filter surfaces may cause poor spore adhesion or loss during transfer [22]. In turn, poor spore adhesion due to low affinity for the filters may result in an underrepresentation of the fungal species present.

To address these limitations, surface chemistry and wettability of filter materials can be tailored to enhance the capture of specific airborne fungal spores. In this study, the surface chemistry and wettability of filter materials were tailored with the aim of specifically capturing different airborne fungal spores. To create modified filters with a desirable range of chemical and wetting properties, we employed plasma polymerization [23]. Unlike other plasma modification options such as corona treatment and oxygen plasma, which have a transient effect [24], plasma‐enhanced chemical vapor polymerization results in a physical coating that changes the surface properties permanently. This technique was selected for surface modification due to its numerous advantages: it enables the one‐step deposition of polymer‐like coatings with a wide range of tunable chemical and physical properties, and it operates without the use of solvents [25]. The coating thickness can be easily controlled and adjusted to as thin as just a few nanometers [26]. A key advantage of plasma‐derived coatings is their ability to adhere to virtually any substrate material. This versatility is particularly valuable, as it allows the desired surface properties to be directly applied to real‐world functional products [27]. The plasma deposition process consists of igniting the vapor phase of an organic monomer into a plasma phase rich in ions, radicals, electrons, photons, etc [28]. These high‐energy species strike the substrate surface, generating reactive sites that enable both chemical and physical bonding. This process leads to strong adhesion of the plasma‐deposited coating [29].

This study explored the application of plasma‐deposited polymer coatings as a surface modification strategy to enhance the spore‐capturing efficiency of conventional air filters. Emphasis was placed on understanding how surface chemistry and coating characteristics influence the interaction between filter surfaces and airborne fungal spores. By utilizing plasma polymerization to functionalize filters with four distinct organic monomers, acrylic acid (AAC), 2‐methyl‐2‐oxazoline (POX), 1,7‐octadiene (OD), and perfluorooctane (PFO), this research systematically investigated the role of different chemical functionalities in promoting spore adhesion and retention. The findings provide valuable insights into the potential of tailored surface modifications to improve bioaerosol sampling in various environmental and clinical settings.

## 2 Results

### 2.1 Surface Characterization

**FIGURE 1:**
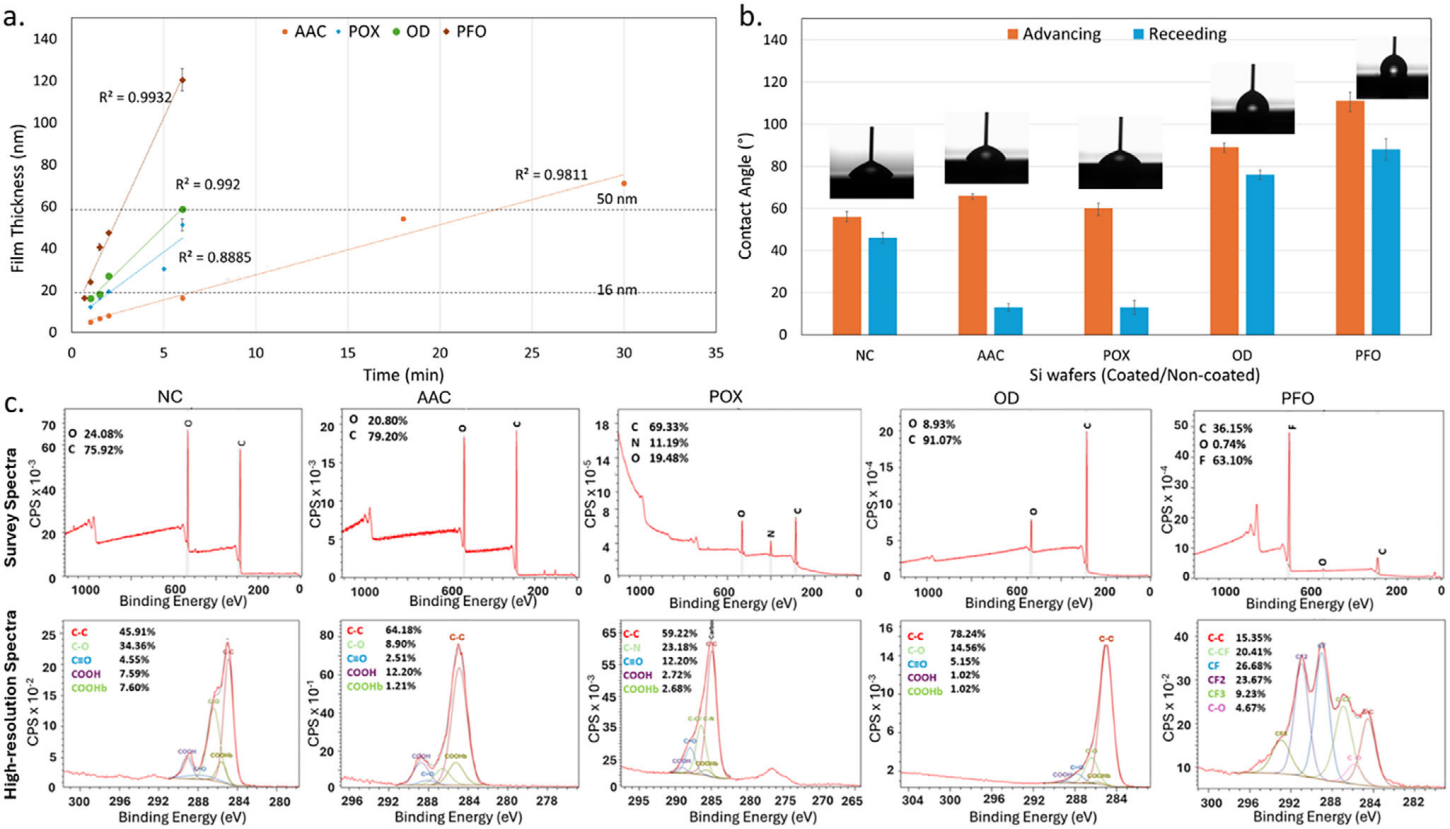
Analysis of AAC, POX, OD, and PFO plasma polymer coatings. (a) Plasma‐deposited film thickness measured by ellipsometry ranges from 16 nm to 75 nm. (b) Advancing and receding water contact angle measurement on non‐coated (NC) and four coated (AAC, POX, OD, PFO) silicon wafers. (c) XPS graphs representing survey and C 1s high‐resolution spectra of coated and non‐coated filters.

The different plasma polymer coatings were characterized through Ellipsometry, XPS, and Contact angle measurement (Figure 1a–c).

#### 2.1.1 Coating Thickness

The thickness of AAC, POX, OD, and PFO plasma polymer films was evaluated by ellipsometry as a function of deposition time (Figure 1a). AAC exhibited the slowest growth, producing 4–50 nm films over 1–30 min. POX and OD grew faster, yielding 12–50 nm and 16–58 nm films, respectively, within 1–6 min. PFO showed the highest deposition rate, forming 16–120 nm films over 40 s to 6 min. Despite the differences in deposition behavior, film thickness consistently increased with longer deposition times, as expected in deposition‐dominant growth regimes [30, 31, 32]. To investigate the potential impact of coating thickness on the capture of airborne fungi, two specific thicknesses 16 nm and 50 ± 5 nm were selected for further evaluation. The corresponding deposition times required to achieve these thicknesses varied for each monomer due to their different deposition rates. The selected times were: 6 min and 18 min for AAC, 1.5 min and 6 min for POX, 1 min and 6 min for OD, and 0.67 min and 2 min for PFO. These conditions were chosen to ensure that all four monomers could be deposited at comparable thicknesses. The blue lines in Figure 1a represent 16 nm and 50 ± 5 nm thicknesses for all monomers.

#### 2.1.2 Coating Surface Chemistry

XPS analysis was performed on filters to determine the coating's atomic composition. Representative survey and high‐resolution XPS spectra, detailing the elemental composition, carbon, oxygen, nitrogen, and fluorine of the different 16 nm thin coatings [non‐coated (NC), AAC, POX, OD, and PFO] are shown in Figure 1c. Different deposition times lead to a film thickness of 16 nm (Figure 1a), hence thicker than the profiling depth of the XPS (approximately 10 nm) [33]. This ensures that the obtained spectra represent just the chemistry of the coatings rather than that of the substrate beneath. Spectra in Figure 1c show fitted survey spectra for each sample with the corresponding elemental compositions in atomic percentages, derived from the integrated peak areas. All substrates contain carbon and oxygen; those coated with POX featured an additional nitrogen peak, whereas PFO coatings exhibited an intense fluorine peak at 688.8 eV, all consistent with the chemical structures of these precursors.

High‐resolution spectra of the C1s region (Figure 1c) provide more insights into the chemistry of the different plasma polymer films. In the following analysis, all the C1s spectra were fitted with five distinct components: C─C (285 eV), C─O (286.5 eV), C═C (288 eV), COOR (289.4 eV), and COORb (285.9 eV). All coatings contained a characteristic peak at 285.0 eV assigned to aliphatic hydrocarbons (C─C, C─H). A distinguishable O─C═O component at 289.2 eV in the spectrum of AAC was assigned to carboxyl acid groups in the coating. The POX films featured a rich chemical functionality, comprising single and double‐bonded nitrogen‐containing groups as well as oxygenated species. These signals are attributed to the presence of amine, amide, imines, nitrile, and intact oxazoline ring [34, 35]. OD contained a main component at 285.0 eV belonging to aliphatic carbons, with smaller C─O and C═O components caused by partial oxidation, which is unavoidable upon exposure to air. In contrast, PFO coating was characterized by the presence of C─CF, CF, CF2, and CF3 at 286.92 eV, 289 eV, 291.0 eV, and 292.9 eV, confirming the film's retention of a perfluorinated structure, which is commonly linked to high hydrophobicity. Also, the presence of a C‐O peak suggests minor contamination or oxidation of the sample surface, which can happen during sample handling or exposure to air.

FTIR shown that the characteristic peaks associated with ester functional groups at ∼1090 cm^−1^, 1240 cm^−1^, and ∼1715 cm^−1^, together with the CH_2_ aliphatic rocking at ∼725 cm^−1^, and the peak at ∼872 cm^−1^ indicative of aromatic C─H bending (terephthalate ring) confirm the filter material is polyethylene terephthalate (PET) (Figure S1) [36].

#### 2.1.3 Coating Wettability

The intrinsic wetting behavior of the different plasma‐polymerized coatings was evaluated using water contact angle measurements on smooth silicon wafers (Figure 1b). The non‐coated (NC) wafer showed hydrophilic behavior (56°). AAC and POX coatings slightly increased the contact angles to 66° and 60°, respectively, remaining within the hydrophilic range. The OD‐coated surface approached hydrophobicity (89°), while the PFO coating produced a distinctly hydrophobic surface (111°). These results align with previously reported trends for similar plasma polymer coatings [34, 37].

### 2.2 Fungal Species Characterization

**FIGURE 2:**
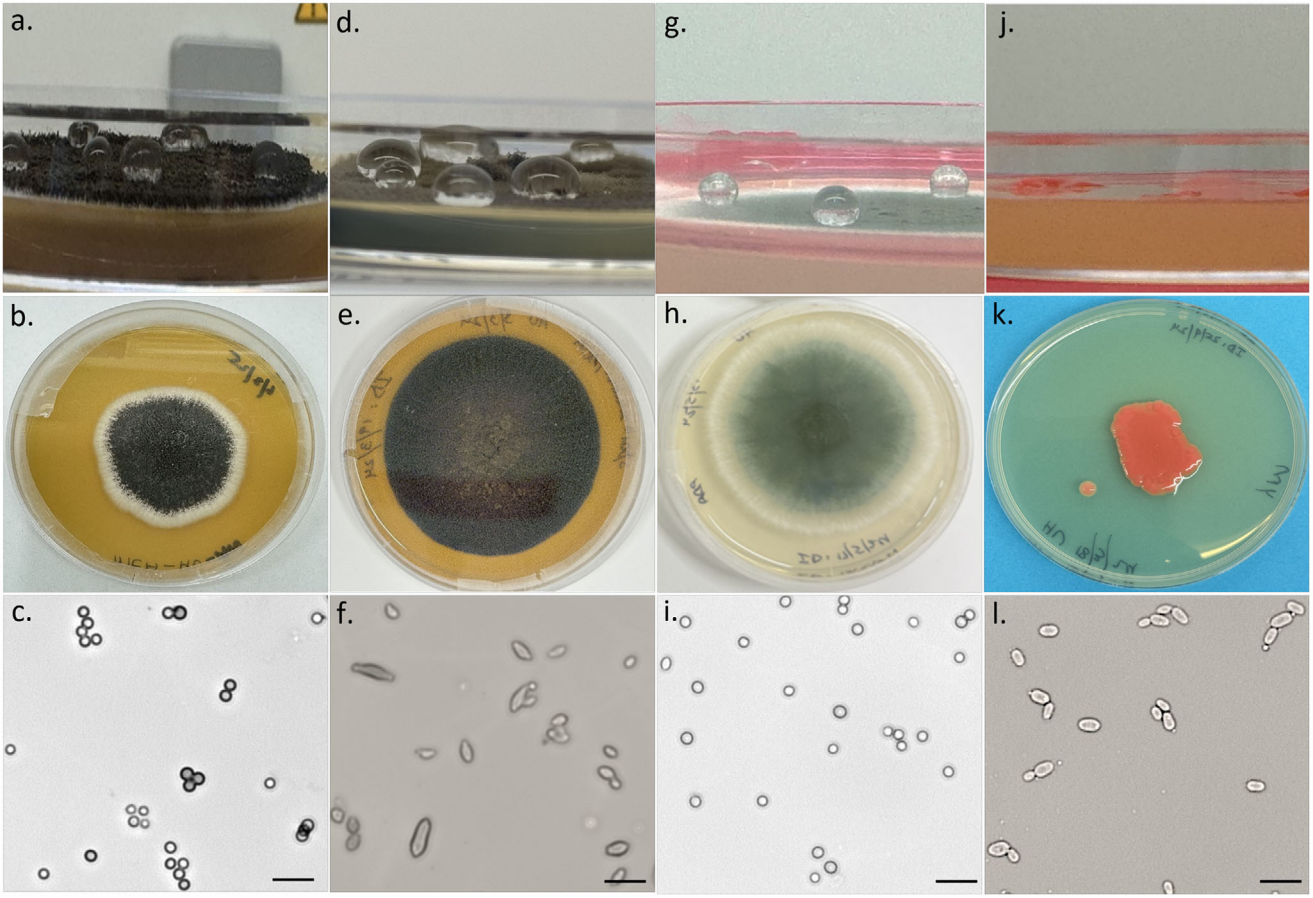
Wettability and morphological characteristics of fungal species, (a–c) *Aspergillus niger*, (d–f) *Cladosporium* sp., (g‐i) *Penicillium roqueforti*, (j‐l) *Rhodotorula glutinis*. Spore images were taken at 400× magnification. The scale bar is 50 µm.

The wettability of fungal spores was assessed by using the water droplet method, the most direct approach, by applying droplets onto the surface of mature sporulating fungal colonies. As shown in Figure 2, the water droplets remained rounded and did not spread across the colony surface, indicating that the fungal colonies of *A. niger*, *Cladosporium* sp., and *P. roqueforti* were hydrophobic. On the other hand, the water droplets spread over the *R. glutinis* colony as shown in Figure 2j, which indicates that this colony is hydrophilic. The apparent wetting properties of the 4 colonies were in good agreement with their chemistry, which was explored via ATR‐FTIR (Figure S2). The FTIR spectra of filamentous species (Figure S2a–c) showed amide bands at ∼1540 cm^−1^ (Amide II) and ∼1650 cm^−1^ (Amide I), as well as a broader Amide III band at ∼1220‐1260 cm^−1^, which likely overlaps with a phosphate contribution. Together with noticeable aliphatic bands at ∼2850 cm^−1^ and ∼2925 cm^−1^ (C─H stretching), these signals are indicative of the presence of proteins (such as hydrophobins) [38]. In addition, polysaccharide‐associated peaks at ∼1030 cm^−1^ and ∼1070 cm^−1^, as well as broad ∼3400 cm^−1^ band from O‐H/N‐H groups were also present. In contrast, in the spectrum collected on *Rhodotorula*, the hydroxyl groups (O‐H) signal in the 3400 cm^−1^ region was the dominant signal and a ∼1640 cm^−1^ peak, likely due to bound water, was also identified together with clear polysaccharide peaks at ∼1030 cm^−1^ and ∼ 1070 cm^−1^, as well as weaker C‐H contributions (Figure S2d).

The morphological characteristics of the isolated fungal species are summarized in Figure 2. *A. niger* formed rapidly growing, circular colonies with regular margins and a compact white mycelial base covered by dense black conidial heads (Figure 2b). Spores were spherical to slightly elliptical (3–5 µm), dark brown to black, and exhibited a rough, echinulate surface (Figure 2c). *Cladosporium* sp. developed slow‐growing, brown to blackish colonies with velvety to powdery surfaces and irregular or feathery edges (Figure 2e). Spores were olive to brownish, oval to cylindrical (2–15 × 2–5 µm), contributing to the colony's dark appearance (Figure 2f). *P. roqueforti* produced fast‐growing, blue‐green colonies with white margins and a velvety surface (Figure 2h). Spores were globose to fusiform (3–6 × 2–4 µm), occurring in long, dry chains (Figure 2i). *R. glutinis* exhibited rapid growth, forming smooth to slightly wrinkled, glossy colonies with color ranging from coral red to salmon (Figure 2k). Spores were ovoid to globose (2–5 × 4–10 µm) with a typical yeast‐like morphology (Figure 2l).

### 2.3 Airborne Spore Capture Analysis

The capture of all four fungal species (*A. niger*, *Cladosporium* sp., *P. roqueforti*, and *R. glutinis*) on NC, [AAC, POX, OD, PFO (16 and 50‐nm coated)] filters was tested using the custom‐made aerosolization chamber described in the methodology section (Aerosolization and sampling of fungal spores).

#### 2.3.1 Optical Microscopy Assessment

**FIGURE 3:**
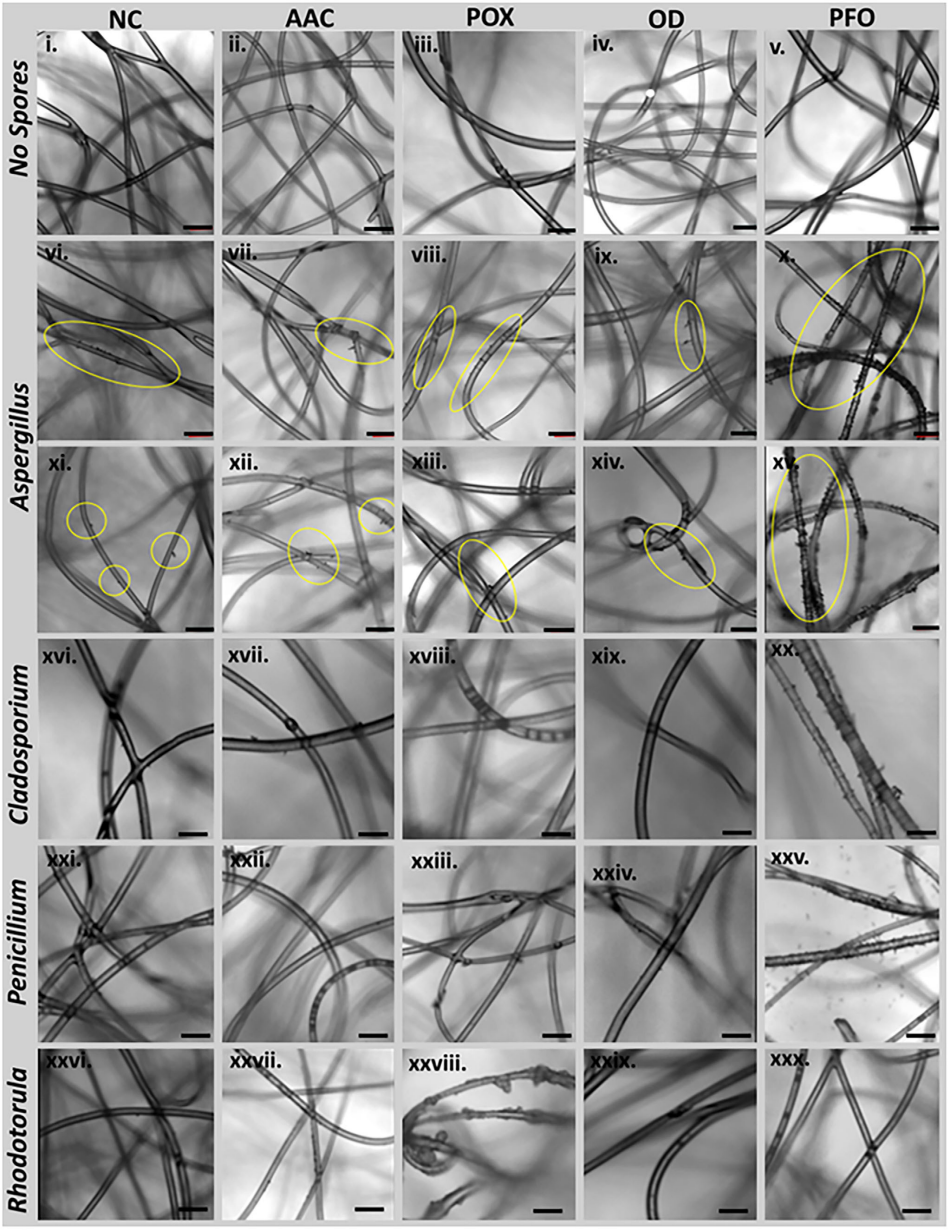
Microscopic images of 4 different fungal species spores captured on 16 nm and 50 nm coated (AAC, POX, OD, PFO), and NC filters at 100x magnification (i‐v) Filters images before sampling with any fungal spores (vi‐x) *Aspergillus niger* spores on 16 nm coated and non‐coated filters (xi‐xv) *Aspergillus niger* spores on 50 nm coated and NC filters (xvi‐xx) *Cladosporium* sp., spores on 50 nm coated and NC filters (xxi‐xxv) *Penicillium roqueforti* spores on 50 nm coated and NC filters (xxvi‐xxx) *Rhodotorula glutinis* spores on 50 nm coated and NC filters. The scale bar is 200 µm.

Optical microscopy shows that PFO‐coated filters exhibited the highest spore capture efficiency for the 3 filamentous fungal species tested, *A. niger*, *Cladosporium* sp., and *P. roqueforti* (Figure 3). Regardless of the coating thickness (16 or 50 nm), PFO coatings promoted stronger adhesion of spores to the filter fibers for all 3 filamentous species, as evident in the microscopic images (Figures S3–S5). In contrast, filters with hydrophilic coatings, namely AAC and POX, captured significantly fewer spores, and the NC filters showed the lowest spore capture. No substantial effect of coating thickness was observed on spore attachment for any of the coatings, suggesting that surface chemistry, rather than film thickness, was the dominant factor influencing spore capture performance.

In contrast, visual inspection of the images showed that the filters that were modified using the POX monomer captured more spores of the yeast *Rhodotorula glutinis*, as compared to other coatings, as shown in Figure 3xxviii). It can be seen in Figure 3xxvii, that few spores were stuck to the AAC‐coated filter, which was slightly less hydrophilic than POX, and no spores were captured on the hydrophobic surfaces, OD and PFO (Figure 3xxix‐xxx). There was no significant effect of coating thickness observed for *Rhodotorula* spores capture, which is evident from the microscopic images of filter fibers (Figure S6).

#### 2.3.2 Electron Microscopy Assessment

**FIGURE 4:**
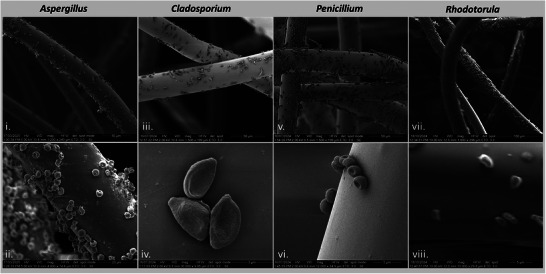
SEM images showing fungal spores captured on filters. (i‐ii) *Aspergillus niger* spores on PFO‐coated filter at (i) 50 µm, 1200× magnification; (ii) 20 µm, 4000× magnification. (iii–iv) *Cladosporium* sp. spores on PFO‐coated filter at (iii) 50 µm, 1500× magnification; (iv) 3 µm, 30,000× magnification. (v–vi) Penicillium roqueforti spores on PFO‐coated filter at (v) 50 µm, 1500× magnification; (vi) 5 µm, 12,000× magnification. (vii–viii) *Rhodotorula glutinis* spores on a POX‐coated filter at (vii) 100 µm, 1000× magnification (viii) 5 µm, 10,000× magnification.

Scanning electron microscopy (SEM) was used as a morphological assessment tool to confirm that the structures attached to the various coated filters are indeed spores, rather than dust or any other contaminants. The SEM images at high magnification (Figure 4ii,iv,vi,viii) reveal sizes and characteristic morphologies consistent with the ones expected for each of the specific spore species, as described in the literature [39, 40, 41]. The spores of *A. niger*, *Cladosporium* sp., and *P. roquefort*i adhered to the PFO‐coated filters, which were hydrophobic (Figure 4i‐vi). Whereas spores of the *R. glutinis* were observed to adhere to the POX‐coated filters, which were hydrophilic (Figure 4vii‐viii). These findings are in agreement with the results from the optical microscopy analysis of the filters described above (Figure 3).

#### 2.3.3 Spore Depth Penetration Tests

**FIGURE 5:**
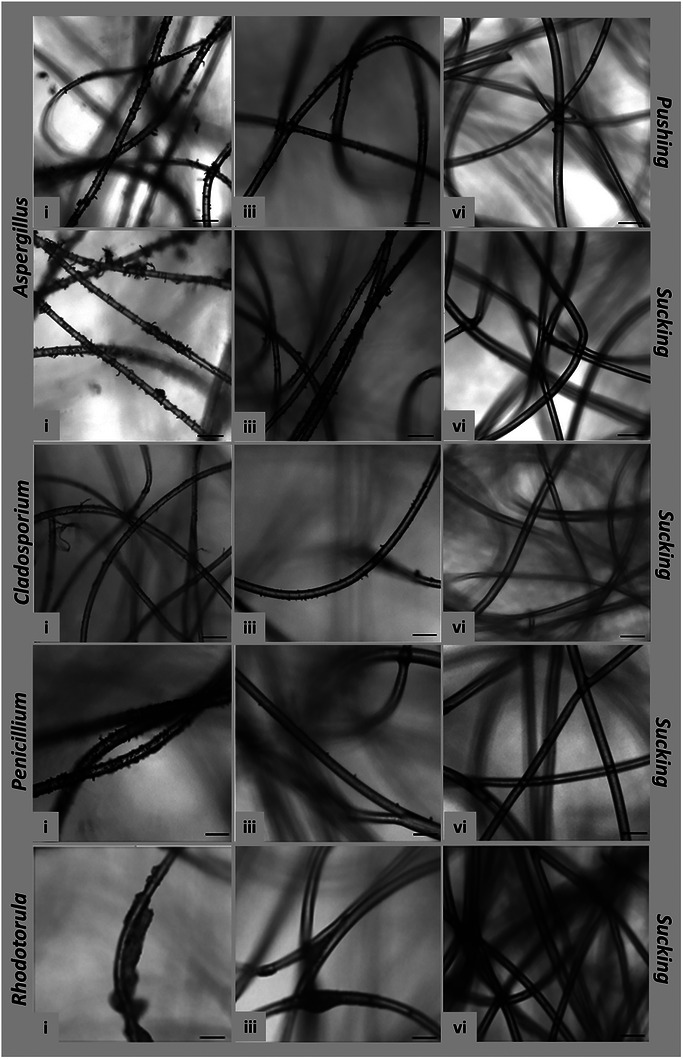
Layer‐wise analysis of filters coated with PFO on both sides with captured *Aspergillus niger*, *Cladosporium* sp., and *Penicillium roqueforti* spores, and POX filters for *Rhodotorula glutinis spores* at 100× magnification. The scale bar is 200 µm.

Cross‐sectional microscopy of PFO‐ and POX‐coated filters was performed to assess fungal spore penetration under bidirectional airflow (17.8 L min^−1^). Across all tested species (*A. niger*, *Cladosporium* sp., *P. roqueforti*, and *R. glutinis*), spores were predominantly retained on the inlet‐facing (i‐layer) surface, with few detected in the intermediate layers and none on the outlet‐facing (vi‐layer) (Figure 5). These findings indicate that, regardless of the airflow direction (pushing or sucking), spore capture occurred mainly through surface‐level retention, with negligible penetration through the filter matrix. Andersen impactor analysis was subsequently used to quantify any spores by passing the filters.

### 2.4 Quantification of Captured Spores

#### 2.4.1 Spore Counts via Hemocytometry

**FIGURE 6:**
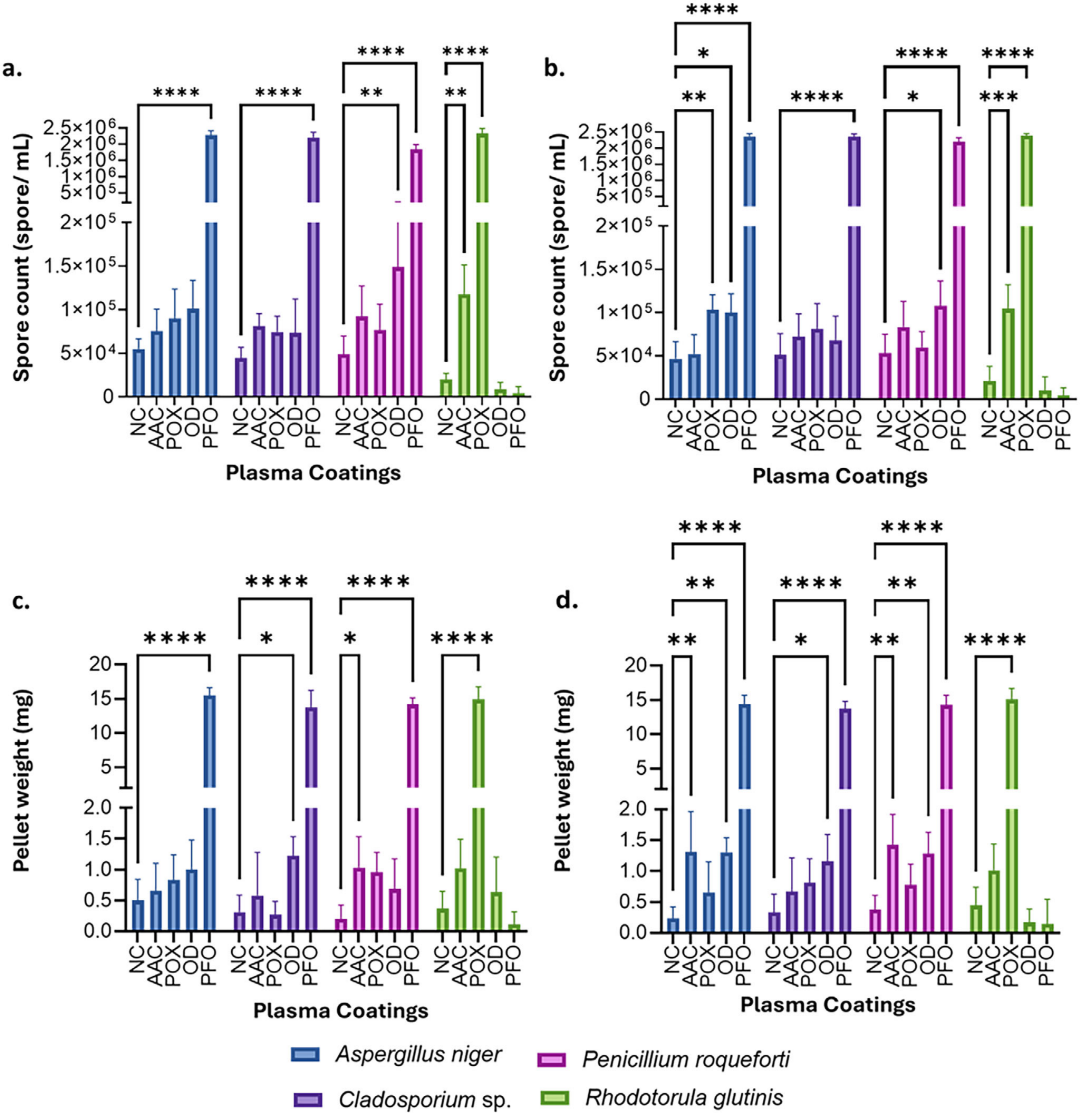
Spore concentration and pellet weight of fungal spores extracted from coated and NC PET filters. (a,b) Spore concentration of four fungal species recovered from filters (a) 16 nm coating (b) 50 nm coating. (c,d) Corresponding spore pellet weights determined from known spore concentrations (c) 16 nm coating (d) 50 nm coating. Significance was determined via two‐way ANOVA with multiple comparisons and uncorrected Fisher's least‐significant difference test. *p*‐value = * ≤0.05, ** ≤0.01, **** ≤0.0001.

The concentrations of spores recovered from NC and plasma‐coated filters (AAC, POX, OD, PFO) as determined via hemocytometry are presented in Figure 6a,b for thin (16 nm) and thick (50 nm) coatings, respectively. For all filamentous fungi (*A. niger*, *Cladosporium* sp., and *P. roqueforti*), the lowest mean spore concentrations were consistently observed on NC filters (≈4 – 5 × 10^4^ spores mL^−^^1^). A gradual increase was noted across AAC, POX, and OD coatings (≈7 × 10^4^ – 1 × 10^5^ spores mL^−^^1^), with the most pronounced effect on PFO‐coated filters, which yielded the highest recoveries (≈2.2–2.3 × 10^6^ spores mL^−^^1^; *p* < 0.0001). This trend was consistent across both coating thicknesses, confirming that increased surface hydrophobicity enhanced capture efficiency for filamentous species. In contrast, *R. glutinis* exhibited the opposite behavior. Hydrophilic coatings (AAC and POX) significantly enhanced spore capture compared to NC (AAC: ≈1 × 10^5^ spores mL^−^^1^, *p* < 0.005; POX: ≈2.2 ‐ 2.3 × 10^6^ spores mL^−^^1^, *p* < 0.0001), whereas OD and PFO coatings showed minimal improvement. Overall, spore capture increased with coating hydrophobicity for filamentous fungi and with hydrophilicity for the yeast species tested in this study, underscoring species‐specific interactions between fungal spores and plasma‐modified filter surfaces.

#### 2.4.2 Spore Mass Quantification

Following hemocytometry quantification, the suspensions were centrifuged, and the resulting spore pellets were weighed to determine the total mass of spores recovered from each filter type (Figure 6c,d). The pellet mass trends closely mirrored the hemocytometer data: PFO‐coated filters yielded the highest spore masses for all filamentous species, while POX‐coated filters exhibited the greatest capture for *R. glutinis*, at both coating thicknesses.

Using this quantification approach, some variations were noted among coatings and thicknesses that were not quantifiable via hemocytometry. The 16 nm AAC‐coated filters produced higher pellet weights (≈1 mg) for *Cladosporium* sp. compared to NC, POX, and OD filters as shown in Figure 6c, while 50 nm AAC coatings also showed elevated pellet masses for filamentous species as shown in Figure 6d. Although this initially suggested possible coating delamination during extraction, control experiments using filters processed without spores produced negligible pellet masses, confirming that the measured mass predominantly originated from spores (Figure S7). Minor contributions from unstable AAC coatings, however, cannot be entirely ruled out.

For filamentous fungi, 50 nm thick OD‐coated filters (the second most hydrophobic filter type) exhibited significantly higher pellet masses than NC filters, suggesting a thickness‐dependent enhancement of spore adhesion not apparent from microscopy alone. Collectively, these quantitative results demonstrate that both coating stability and film thickness influence spore capture and highlight the distinct responses of filamentous and yeast species to surface hydrophobicity.

Recovery efficiency of the vortex elution step was evaluated by comparing the amount of fungal biomass recovered after PBS vortex elution to the total biomass captured on the filters. Across all species and filter types, recovery was consistently high when sufficient biomass was collected, with values ≥98% under high‐capture conditions where the recovered mass was well above the 0.1 mg balance precision. Greater variation was observed only when very low biomass was captured, typically <0.5 mg, where small weighing differences are amplified in the percentage recovery calculation. These values therefore indicate efficient vortex elution overall, with low‐mass variability largely reflecting measurement precision rather than coating‐dependent recovery effects (Table S1).

### 2.5 Filters Geometry Tests

To assess whether filter geometry influences coating performance, POX and PFO coatings previously shown to enhance spore capture were deposited on flat EPM2000 membranes (0.3 µm pore size), with NC membranes serving as controls. Only thin (16 nm) plasma polymer films were evaluated for this comparison.

**FIGURE 7:**
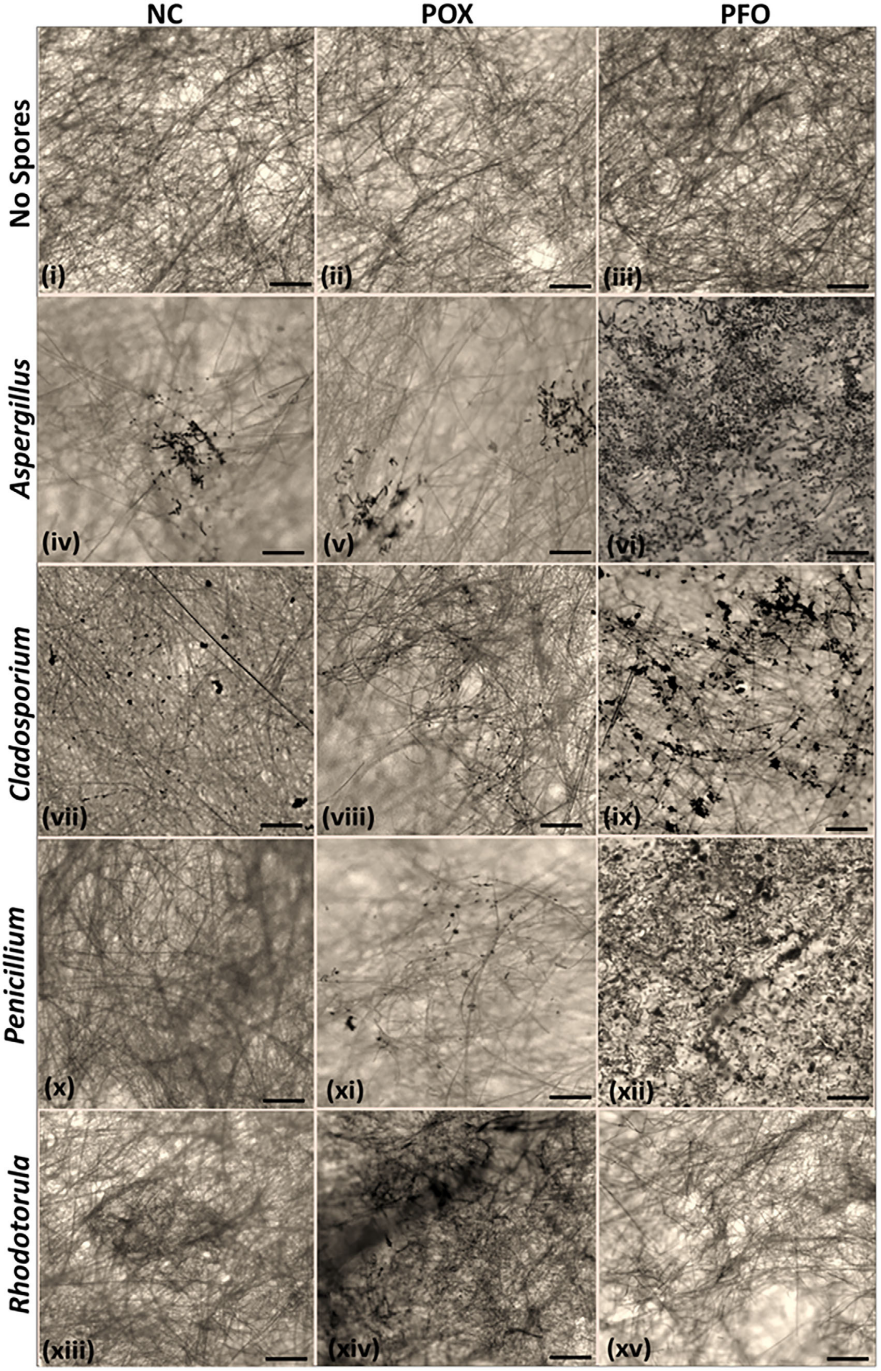
Microscopic images of fungal spores captured on EPM2000 membranes at 100x magnification. Columns showing NC, POX, and PFO filters. Rows showing different fungal species spores captured on filters. The scale bar was 200 µm.

Microscopic findings revealed trends consistent with observations made on the thicker filters: NC membranes retained the fewest spores, POX‐coated membranes showed moderate improvement, and PFO‐coated membranes exhibited dense spore retention, particularly for filamentous fungi (*A. niger*, *Cladosporium* sp., *P. roqueforti*) as shown in Figure 7vi,ix,xii. While the quantitative measurement performed on the spores recovered from EPM2000 membranes confirms those trends (Figure S8), the absolute values of spore counts and pellet weight may not be representative of the membrane's true efficiency because practical sealing issues were identified in the subsequent Andersen cascade efficiency tests, as discussed in the following sections.

### 2.6 Filter Efficiency and Spore Loss

**FIGURE 8:**
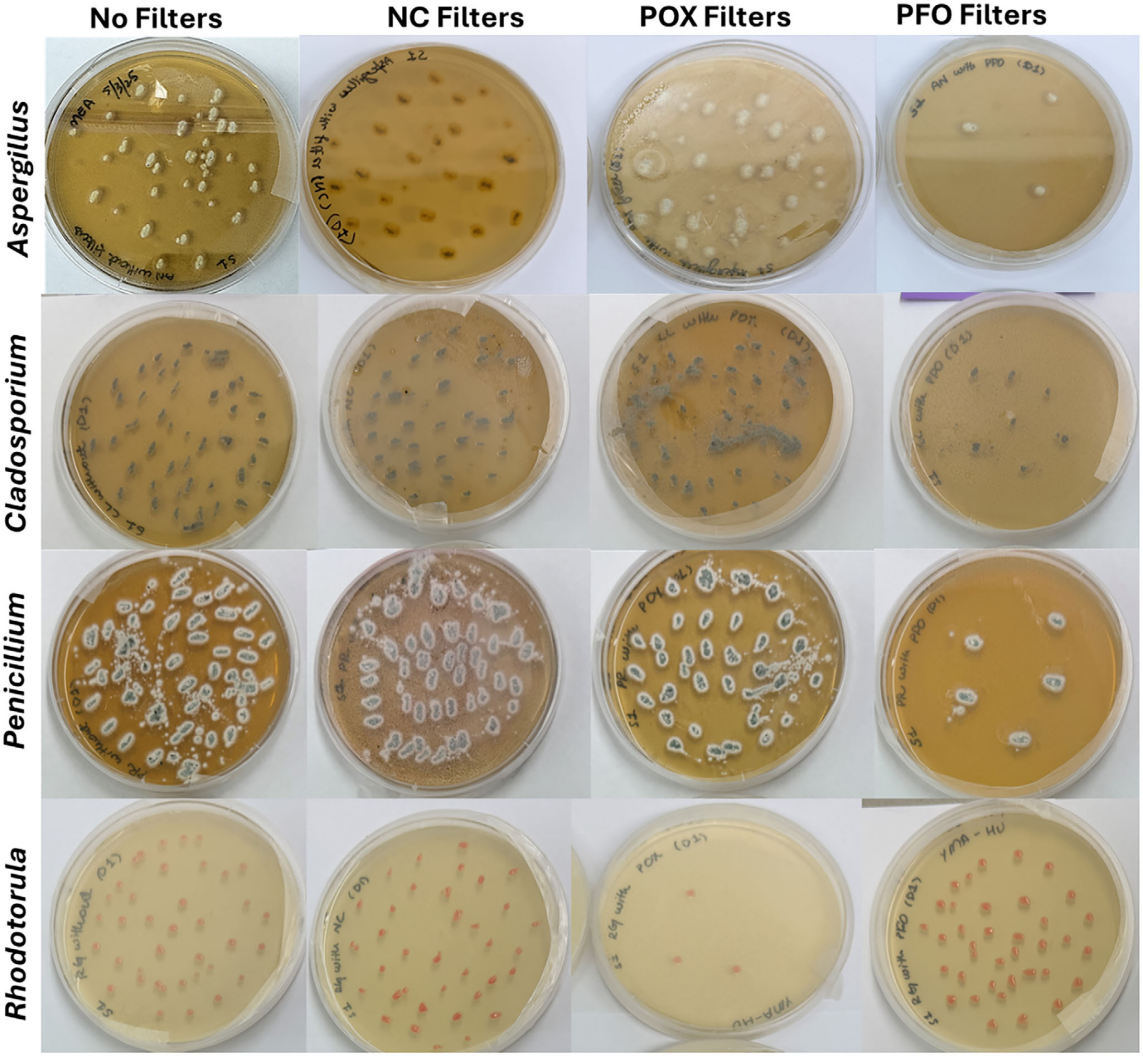
CFU counting on Agar plates for 4 different fungal species after using Andersen Sampler along with experimental setup, without filters and with PET filters (NC, POX, PFO).

To evaluate the performance of the custom air sampling system and to determine the spore capture efficiency of coated and non‐coated PET filters and EPM2000 membranes, an Andersen cascade impactor was used. Colony‐forming units (CFUs) were enumerated after 3 days of incubation at 26°C on MEA for *A. niger*, *Cladosporium* sp., and *P. roqueforti*, while YMA was used for *R. glutinis* spores, as shown in Figure 8.

**FIGURE 9:**
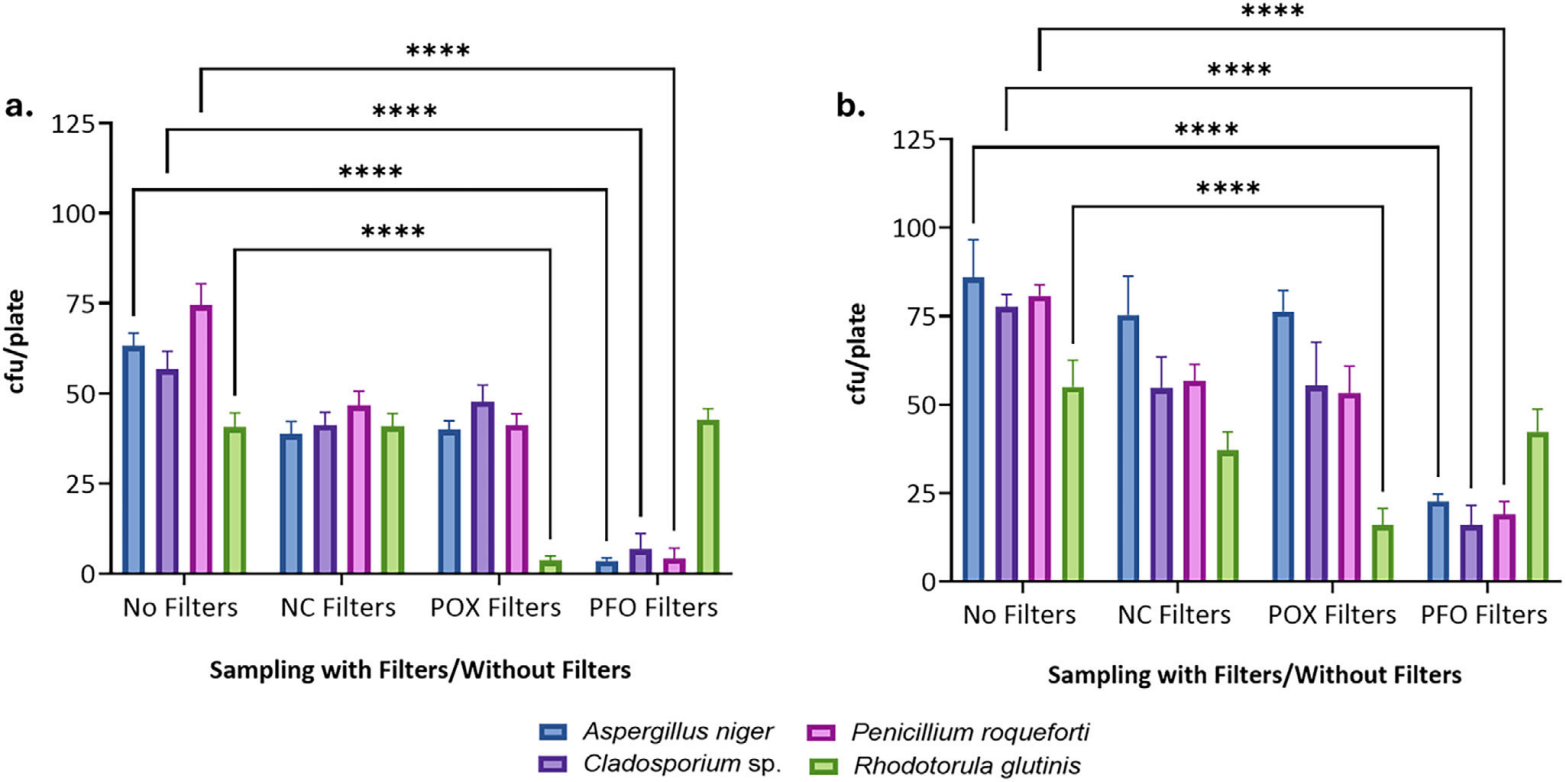
Total fungal spore capture (CFU/plate) on coated (POX, PFO) and non‐coated (NC) surfaces (a) PET filters (b) EPM2000 membranes. Significant differences in capture efficiency were observed among filter types. Data represent mean ± SD. Significance was determined via two‐way ANOVA with multiple comparisons and uncorrected Fisher's least‐significant difference test. *p*‐value = * ≤0.05, ** ≤0.01, **** ≤0.0001.

As shown in Figure 9a, in the absence of any filter present between the culture plate and Andersen sampler, *A. niger* produced 63 cfu plate^−1^, which decreased to ∼38 cfu plate^−1^ with NC filters in line, corresponding to ∼40% capture efficiency. POX‐coated filters showed a similar performance (∼40 cfu plate^−1^), consistent with the weak interaction between *A. niger* spores and this coating. In contrast, PFO‐coated membranes reduced the colony count to ∼4 cfu plate^−1^, corresponding to ∼95% capture efficiency (*p* < 0.0001). A similar pattern was observed for *Cladosporium* sp. and *P. roqueforti*. Under control conditions (no filter), 56 and 74 cfu plate^−^^1^ were recorded, respectively. NC and POX‐coated filters achieved 27–39% and 15–46% retention, whereas PFO‐coated filters reached 88% for *Cladosporium* and 94% for *Penicillium*, indicating significantly improved performance (*p* < 0.0001). The slightly lower efficiency for *Cladosporium* may reflect species‐specific surface interactions.

For *R. glutinis*, control plates showed a lower baseline count (40 cfu plate^−^^1^), consistent with its reduced aerosolization capacity. Neither NC nor PFO filters captured measurable amounts of spores, whereas POX‐coated filters achieved 90% retention. Despite the known lipid‐rich and reportedly hydrophobic nature of *R. glutinis* [42], no adhesion to PFO‐coated surfaces was observed, indicating that other factors influence the yeast‐surface interaction in the conditions tested here.

Similarly, the capture efficiency of coated and non‐coated EMP2000 membranes was tested to evaluate the spore loss from the system. Control experiments, conducted in the absence of any membranes, established baseline CFU values for each species.

For *A. niger*, 86 cfu plate^−^^1^ were recorded under control conditions, which was higher than the baseline observed in the previous test (63 cfu plate^−1^) due to the difference in airflow rate. The counts decreased to 75 and 76 cfu plate^−^^1^ with NC and POX membranes, corresponding to 13% and 12% capture efficiency, respectively. PFO‐coated membranes significantly reduced the count to 22 cfu plate^−^^1^, achieving 75% capture (*p* < 0.0001) as shown in Figure 9b. Comparable trends were observed for *Cladosporium* sp. and *P. roqueforti*. Control counts were 77 and 80 cfu plate^−^^1^, respectively, with NC and POX membranes capturing only 29–33% of spores. In contrast, PFO‐coated membranes demonstrated capture efficiencies of 79% for *Cladosporium* and 76% for *Penicillium*, which were lower than those observed previously for PET filters (Figure 9a). For *R. glutinis*, baseline counts were lower (55 cfu plate^−^^1^), and NC and PFO membranes showed negligible retention, while POX membranes achieved ∼71% capture.

Although EPM2000 membranes possess a defined pore size of 0.3 µm, their captured efficiency is lower than that of the PET filter (Figure 9), and the spore count and spore mass measured on the thin membranes were comparable to those recorded on the thick filters, not higher (Figure S8). This result is counterintuitive, as smaller pore sizes were expected to improve spore retention through size exclusion. We hypothesize that because the thin NC membranes were here mounted in a filter holder primarily designed for depth‐type filters, small gaps could potentially form at the membrane edges, allowing airflow and spores to bypass the filter surface. A sub‐optimal filter seal could indeed explain the lower‐than‐expected capture efficiency recorded.

Overall, PFO coatings consistently provided the highest retention for filamentous fungi, whereas POX coatings were most effective for yeast‐like spores tested here, confirming species‐dependent interactions with plasma‐modified surfaces, regardless of the filter's intrinsic geometry.

A positive‐hole correction was applied to the CFU data generated from the six‐stage Andersen cascade impactor. This correction accounts for the likelihood that multiple spores can pass through the same hole during sampling, even though only a single colony can form at the corresponding location of the agar plate [43]. Without correction, this overlap leads to an underestimation of the true airborne spore concentration. After applying the positive‐hole adjustment, the corrected values were not statistically different from the uncorrected counts (Figure S9).

## 3 Discussion

### 3.1 Effect of Deposition Rates and Thickness Range and Substrate Dependence

Plasma polymer films showed varying deposition rates and thickness ranges depending on the monomer used. Despite these differences, all monomers exhibited a clear trend of increasing film thickness with longer deposition times, consistent with previous studies on comparable plasma polymer coatings [30, 31, 32]. The variation in growth rates likely reflects differences in monomer properties, such as fragmentation tendencies, as well as deposition conditions, including power, pressure, and flow rate [32]. These results highlight the critical role of both chemical and processing parameters in tailoring plasma polymer film growth.

Plasma polymer studies have demonstrated that the underlying substrate only influences the growth rate, morphology and chemistry in the first few nanometers (1 to 5 nm) of the plasma polymer film [26, 27]. Here, the targeted thicknesses are above this threshold—16 nm and 50 nm—therefore, all results for plasma polymer coatings deposited on various substrates (silicon wafers, PET) discussed in this text are essentially substrate‐independent.

### 3.2 Effect of Wettability

To investigate the surface properties of our fungal species, we first assessed the wettability of fungal colonies. Interestingly, *Rhodotorula glutinis* exhibited a more hydrophilic surface compared to *Aspergillus, Cladosporium*, and *Penicillium* spores (Figure 2j), indicating that water droplets adhered more readily to *Rhodotorula* colonies.

The observed difference in wettability between *Rhodotorula* and the remaining three colonies can be explained by the nature of the fungal structures themselves. Filamentous fungi such as *Aspergillus* and *Penicillium* produce thick‐walled, dehydrated spores enriched with hydrophobins in their cell walls. These amphiphilic proteins self‐assemble into structured rodlet layers at hydrophilic‐hydrophobic interfaces, forming amphipathic layers that reduce wettability, enable aerial dispersal, and mediate adhesion to hydrophobic surfaces such as plant cuticles [44, 45]. Hydrophobins also play a key role in biofilm formation, helping fungi establish stable colonies capable of withstanding environmental stress [46]. More broadly, spore surface chemistry in filamentous fungi consists of an inner polysaccharide wall and a species‐specific outer layer of proteins and pigments. Many filamentous fungi, including *Aspergillus* and *Penicillium*, coat spores and aerial hyphae with hydrophobin, forming ∼10 nm rodlet fibrils that create a highly hydrophobic surface, similar to the super hydrophobicity observed on certain plant leaves, which have highly crystalline waxes on their surfaces. This coating renders the spores non‐wettable and masks underlying glucans and other cell wall carbohydrates [47, 48, 49, 50, 51]. In *A. niger* and closely related species, melanin in the outer layer contributes to hydrophobicity and adhesion [52, 53]. In contrast, *Rhodotorula glutinis* demonstrated a more hydrophilic surface compared to *Aspergillus, Cladosporium*, and *Penicillium* spores (Figure 2j). Previous studies have reported a strong dependence of the wetting properties of *Rhodotorula* yeast cells on the growth stage they are at the time of wettability assessment [54]. During initial growth phases, they were found to exhibit hydrophobic behavior, while at later growth stages, consistent with mature sporulating colonies like the colonies studied here, a significant drop in the cell surface hydrophobicity (CSH) by about 50% was observed. *Rhodotorula* colonies primarily consist of blastoconidia, the budding yeast cells with relatively thin walls and a moist, metabolically active surface, which favors hydrophilic characteristics that facilitate nutrient exchange [55, 56]. Therefore, the exposed polysaccharide‐rich components of *Rhodotorula* and extracellular materials likely contribute to the observed hydrophilicity of the mature colony (Figure 2j), in contrast to the hydrophobicity demonstrated by the dehydrated, hydrophobin‐rich spores of *Aspergillus, Cladosporium*, and *Penicillium*.

To further correlate the wettability results with the nature of the fungal structures, the surface chemistry of the spores and yeast cells was studied with infrared spectroscopy (Figure S2). The ATR‐FTIR analysis suggests *Aspergillus*, *Cladosporium*, and *Penicillium* spores have a similar and more protein‐rich surface (see nitrogen‐associated bands in Figure S2a–c) than *Rhodotorula* yeast cells. The presence of amide and aliphatic bands in the filamentous spores indicates a protein‐rich surface with hydrophobic characteristics, consistent with the non‐wetting behavior observed in the wetting studies (Figure 2a,d,g). In contrast, the strong hydroxyl signal and bound water peak observed for *Rhodotorula glutinis*, together with polysaccharide‐associated features, indicate a more hydrated, polysaccharide‐rich surface, consistent with its hydrophilic behaviour (Figure 2j).

### 3.3 Hydrophobic‐Hydrophobic Interactions

The enhanced spore‐capturing efficiency of PFO‐coated filters can be attributed to strong chemical and physical compatibility between the filter surface and the structural characteristics of the fungal spores. Unlike other coatings, PFO is chemically inert, neutral or minimally charged, and also forms a highly hydrophobic and non‐polar surface due to the presence of perfluorinated carbon chains (C_8_F_16_), which repel water and reduce surface energy [57]. As discussed above, fungal spores such as *Aspergillus niger*, *Cladosporium* sp., and *Penicillium roqueforti* possess hydrophobic outer spore layers. This is composed of proteins like hydrophobin and surface pigments such as melanin, which make it both hydrophobic and negatively charged [58, 59].

In an airborne environment, hydrophobic surfaces tend to avoid contact with water, and when the PFO‐coated surface and spore surface encounter each other, they preferentially adhere to minimize interfacial energy. This occurs because forming an interface between a hydrophobic surface and water is energetically unfavorable: the system must disrupt the hydrogen‐bonding network of water to accommodate a surface that cannot engage in such interactions. This increases the interfacial free energy, so the system minimizes contact to reduce its overall surface energy.

This entropy‐driven hydrophobic‐hydrophobic interaction becomes the dominant adhesion mechanism. The PFO‐coated surface provides a favorable surface where hydrophobic spores can stably adhere. The absence of significant surface charge on PFO also means that electrostatic repulsion is minimized, allowing spores to interact with the filter through van der Waals forces and non‐polar interactions. This overall compatibility encompassing hydrophobic interaction, weak intermolecular attractions, and charge neutrality explains the increased adhesion of filamentous fungal spores to PFO‐modified filters, as observed in Figure 2a,d,e.

### 3.4 Electrostatic Interactions and Hydrogen Bonding

The enhanced adhesion of *R. glutinis* spores to filters treated with the POX monomer is likely attributed to the chemical groups introduced by the coating. The hydroxyl (─OH) and amide (─NH) groups render the filter surface both hydrophilic and polar. The spore cell wall of *R. glutinis* is rich in polysaccharides and glycoproteins, including mannose‐containing structures, which provide multiple sites for polar and hydrogen‐bonding interactions with surfaces [60]. These interactions likely facilitate cross‐linking with the functional groups of the POX monomer, promoting stronger attachment. In addition to these polar interactions, cell‐wall glycoproteins and associated carbohydrates can mediate adhesion through other weak forces, such as hydrogen bonding and van der Waals interactions, with hydrophilic and polar surfaces [61]. Overall, the attachment of *R. glutinis* spores to POX‐coated filters appears to result from a combination of hydrophilic interactions, chemical affinity between surface groups, electrostatic forces, and the physical characteristics of the coated surface, all of which create an environment conducive to effective spore adhesion.

The experimental results demonstrate that spore adhesion to coated filters is largely governed by the compatibility between the surface chemistry of the filter and the physicochemical properties of the fungal spores. PFO coatings significantly enhanced the capture of hydrophobic filamentous spores (*Aspergillus niger*, *Cladosporium* sp., and *Penicillium roqueforti*), regardless of coating thickness, due to favorable hydrophobic and non‐polar interactions. In contrast, hydrophilic coatings like POX selectively enhanced adhesion of hydrophilic yeast spores such as *Rhodotorula glutinis*, likely due to polar interactions, electrostatic affinity, and the presence of functional groups capable of forming hydrogen bonds or cross‐linking with extracellular polysaccharides. These findings highlight the importance of tailoring filter surface chemistry, rather than coating thickness, to match the surface properties of target airborne fungal species spores for improved capture efficiency.

### 3.5 Influence of Filter Geometry

The capture efficiency of EPM2000 membranes was tested because, unlike the PET filters, they are flat and possess a defined pore size of 0.3 µm. However, the interpretation of their filtration performance was here limited by technical challenges associated with achieving reliable sealing during sampling. Poor sealing may permit airflow bypass at the membrane edges, leading to potential underestimation of spore retention capacity. As such, the absolute values obtained from these experiments could not be used to draw quantitative conclusions on intrinsic capture performance. Despite this limitation, qualitative observations confirmed trends broadly consistent with those observed for thicker PET fiber filters, suggesting that coating‐dependent behavior was preserved across different geometries. It is also worth noting that filtration performance in aerosolized biological systems is governed by a complex interplay of physical and chemical mechanisms rather than pore size alone. Under dynamic airflow conditions, additional forces such as inertial impaction, interception, diffusion, and electrostatic attraction/repulsion significantly influence particle capture [62]. Consequently, filters that theoretically possess sufficient pore tightness may still allow partial passage or instead rebound of fungal spores if surface interactions are unfavorable. Conversely, thick filters with larger nominal pore size may efficiently trap spores within the depth of their network. Such a phenomenon could also, in part, explain the differences seen between the EPM2000 membrane and PET filters.

Overall, while these findings provide preliminary insight into the influence of filter geometry, the superior performance of plasma‐coated membranes compared to unmodified NC filters across different filter types reinforces that surface chemistry ‐more so than pore size alone‐ plays a dominant role in governing fungal spore capture. This highlights the critical role of surface modification in improving both the quantitative and qualitative reliability of fungal spore collection from air samples.

## 4 Conclusion

Enhancing indoor air quality has significant implications for human health, building safety, and environmental monitoring, highlighting the need for efficient and selective capture of airborne fungal spores. Unlike conventional size‐exclusion filters, which rely solely on pore size to trap particles, the filters employed in this study leverage surface chemistry to selectively retain fungal spores. This selectivity is particularly important, as spores vary widely in size, shape, and surface properties, and traditional filters may either fail to capture certain spores or disproportionately retain others. The findings of this study demonstrate that plasma‐deposited polymer coatings enhance fungal spore capture by tailoring filter surface chemistry. Filters coated with acrylic acid (AAC), 2‐methyl‐2‐oxazoline (POX), 1,7‐octadiene (OD), and perfluorooctane (PFO) were characterized by ellipsometry, XPS, and contact angle analysis, revealing that surface chemistry, not coating thickness, governs capture efficiency. Spore adhesion was species‐specific, reflecting hydrophobic or hydrophilic interactions between spores and coated surfaces. PFO coatings effectively captured hydrophobic spores (*Aspergillus niger*, *Cladosporium* sp., *Penicillium roqueforti*), while hydrophilic POX coatings were optimal for *Rhodotorula glutinis*. Results were consistent across microscopy, spore counts, pellet mass, and Andersen sampler data. Coating both sides and varying filter geometries confirmed that plasma modification operates independently of structure or flow direction. Overall, plasma polymerization offers a scalable, solvent‐free strategy to engineer species‐selective filters for bioaerosol monitoring and downstream molecular analyses. This proof‐of‐concept study used single‐species aerosols to investigate the performance of plasma polymer coating in controlled conditions. While this approach provides mechanistic insight, real‐world bioaerosols are typically composed of multiple species. Future studies should therefore assess filter performance under mixed‐species conditions to better reflect environmental complexity.

## 5 Materials and Methods

### 5.1 Materials

#### 5.1.1 Filters and Chemicals

Synthetic HVAC filter material was obtained from RS Components, Australia, with the following specifications: Pad filter, Grade G4, and 20 mm nominal depth were used (RS Stock number 122–1774). The material was cut to size using scissors prior to use. The filter fibers were composed of Polyethylene terephthalate (PET) and arranged into a gauze‐like material. The filter's geometry results in nonuniform pore sizes, generally ranging from 5 µm to 15 µm, through which air passes between the fibers. Whatman EPM 2000 air sampling filters (CAT no. 1882‐047) with a pore size of 0.3 µm were purchased from Cytiva Australia. Four monomers, purchased from Sigma‐Aldrich and Merck, were used as precursors for plasma polymer deposition, namely Acrylic acid (AAC), 2‐Methyl‐2‐oxazoline (POX), 1,7‐Octadiene (OD), and perfluorooctane (PFO), with purities of 99%, 98%, 98%, and 98%, respectively Table 1. Silicon wafers (made in Japan, D&X Co., Ltd) were purchased from the Australian National Fabrication Facility and used as a reference material.

**TABLE 1:** List of liquid precursors used for the plasma polymer coatings, along with their respective manufacturers, density, molecular weight, purity, and wettability.

Monomer (acronym)	Chemical structure	Polarity	Wettability (resulting coating)
Acrylic Acid (AAC)	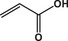	Polar	Hydrophilic
2‐Methyl‐2‐oxazoline (POX)	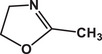	Moderately polar	Amphiphilic
1,7‐Octadiene (OD)	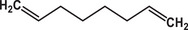	Non‐polar	Hydrophobic
Perfluorooctane (PFO)	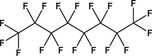	Non‐polar	Highly hydrophobic

### 5.2 Methods

#### 5.2.1 Plasma Deposition

The filters were coated with a nanothin polymeric using plasma modification as follows. Plasma polymers were deposited on the filters using a custom‐made plasma reactor (Figure S10), the details of which have been previously outlined [63]. The plasma was ignited by using a 13.56 MHz radio frequency (RF) generator coupled to a matching network (Coaxial Power System). Before the plasma deposition, the sample substrates, including filters as test materials and silicon wafers as controls, were introduced into the plasma reactor and primed at 30 W, for 3 min under an air plasma at 1.2 × 10^−1^ mbar. The reactor was pumped down to a base pressure of 2 × 10^−2^ mbar to eliminate atmospheric nitrogen and oxygen from the chamber, with pressure being monitored using a Pirani gauge (APG100, Edwards) and controlled by a needle valve (Chell Instrument, UK). The different monomers, AAC, POX, OD, and PFO, were introduced into the plasma chamber in the gaseous form at ignition pressures of 0.15, 0.12, 0.13, and 0.20 mbar, respectively Table 2. The deposition power ranged from 10 W to 30 W, and the duration varied from 40 s to 30 min to achieve the desired film thickness according to previously published procedures [33, 64, 65]. Triplicate substrates were used for all material characterization and fungal spore capture.

**TABLE 2:** Summary of plasma polymer deposition conditions for different coatings.

Monomer	Ignition Pressure (mbar)	RF Power (W)	16 nm Deposition time (min)	50 nm Deposition time (min)
Acrylic Acid (AAC)	0.15	10	6 min	18 min
2‐Methyl‐2‐oxazoline (POX)	0.12	30	1.5 min	6 min
1,7‐Octadiene (OD)	0.13	20	1 min	6 min
Perfluorooctane (PFO)	0.2	20	0.67 min	2 min

### 5.3 Surface Characterization

#### 5.3.1 Ellipsometry

The thickness of the plasma polymer film was determined using an ellipsometer (VASE, J.A. Woollam). Experimental data were collected over a wavelength range of 250 to 1100 nm at incident angles of 65° and 75°. The thickness of the plasma polymer layer deposited on a pristine silicon wafer was determined using a well‐established Cauchy model and fitting procedure [66]. Specifically, a three‐layer Cauchy model was utilized, comprising an infinitely thick silicon substrate with a 2 nm native oxide layer, overlaid by the plasma polymer film under investigation. The selection of the Cauchy model was based on its suitability for describing the optical properties of plasma polymer films [33]. The optical parameters for the silicon substrate and native oxide layer were extracted from the WVASE32 software library [67].

#### 5.3.2 X‐ray Photoelectron Spectroscopy

X‐ray photoelectron spectroscopy (XPS) analysis provides information on the atomic composition of the films’ topmost surface, down to a few nanometers beneath the plasma‐deposited polymer layer. Survey spectra were acquired to determine the respective amount of the elements present on the surface (in %), and high‐resolution C 1s spectra were collected to examine the carbon chemical bonding environment. XPS analysis was conducted on a Kratos Axis Ultra DLD spectrometer using a monochromatic Al Kα X‐ray source operating at 225 W, corresponding to an energy of 1486.6 eV. The area of analysis was 0.3×0.7 mm. An internal flood gun was employed to minimize the charging of the samples. Survey spectra were collected at a dwell time of 55 ms with 160 eV pass energy with three sweeps. High‐resolution C 1s spectra were collected with a pass energy of 20 eV and a step size of 0.1 eV. Data analysis was performed using CasaXPS (Version 2.3.24, Casa Software Ltd, UK) [68]. The spectra were corrected for sample charging effects by setting the C─C aliphatic hydrocarbon signal to 285.0 eV. The peak fitting was carried out by applying a Shirley background and Gaussian‐Lorentzian functions (GL50).

#### 5.3.3 Infrared Spectroscopy

Infrared spectrum of the PET filter material was also collected under vacuum on a Bruker Vertex 80v FTIR spectrometer using a diamond attenuated total reflectance (ATR) attachment. 50 scans at a resolution of 2 cm^−1^ were averaged.

Thermo Fisher spectrometer Nicolet iS50 with an ATR attachment with a diamond crystal was also used to characterize the surfaces of the spores. Background and sample spectra were collected at 64 scans with 2 cm^−1^ resolution at a spectral range 4000 to 500 cm^−1^. The spores for analysis were collected from the top surface of mature sporulating colonies and did not undergo any drying.

#### 5.3.4 Contact Angle Measurement

Contact angle measurement, including advancing and receding angles of water droplets on silicon wafers, was carried out using an optical goniometer (OCA 25, Data Physics). This characterization technique was used to evaluate the surface wettability of the coatings. The surfaces studied included uncoated and plasma polymer‐coated silicon wafers. All measurements and readings were conducted in a sessile drop configuration and analyzed with SCA20 software (DataPhysics, Germany). Droplets of 2.5 to 5 µL were used on every substrate, with water added at a controlled rate of 0.05 µL/s via a Hamilton syringe. When the three‐phase contact line was set in motion, the profile of the droplet was automatically extracted by the software, and using the tangent method, the advancing and receding water contact angles were measured [69].

#### 5.3.5 Wettability of Mature Fungal Colonies

To test the wettability of fungal spores, 30 µL of Milli‐Q water was placed on the surface of mature sporulating fungal colonies using a micropipette. The angle formed between the liquid droplet and the fungal colony surface was observed from the side using an iPhone (15 Pro Max) phone camera to take images. Multiple measurements across a minimum of 3 different areas of the colony were taken to observe the stability of water, as wettability can vary depending on growth patterns.

### 5.4 Fungal Colonies Culture

Four fungal species were used in this study: *Cladosporium* sp. (Environmental Health Lab, Flinders University), *Aspergillus niger* (ATCC 6257), *Penicillium roqueforti* Thom (ATCC 10110), and *Rhodotorula glutinis* (Fresenius) Harrison (ATCC 32765) obtained from American Type Culture Collection (Manassas, VA, USA). Growth media included Malt Extract Agar (MEA, CM0059B) and Potato Dextrose Agar (PDA, CM01390) from Thermo Fisher Scientific (Australia), and Yeast Malt Agar (YM Agar, Y3127) from Merck Sigma‐Aldrich (Australia).

For media preparation, 50 g MEA, 39 g PDA, or 41 g YM agar were each dissolved in 1 L of deionized water, autoclaved at 121°C for 15 min (fluid cycle), cooled, and poured into Petri dishes. Plates were left overnight in a biosafety cabinet to prevent condensation. *Aspergillus niger* and *Cladosporium* sp. were cultured on MEA, *P. roqueforti* on PDA, and *R. glutinis* on YM agar. Incubation was carried out at 26°C for 5–7 days (*A. niger*, *P. roqueforti*, *R. glutinis*) and 18–20 days (*Cladosporium* sp.) to obtain mature sporulating colonies.

### 5.5 Aerosolization and Sampling of Fungal Spores

Fungal spores were aerosolized in a custom‐built chamber (Figure S11) placed within a Class II biosafety cabinet. Mature sporulating colonies on Petri dishes served as spore sources. N_2_ gas was passed through the chamber at a constant flow rate of 17.8 L min^−^^1^ and 1.5 bar pressure, regulated by a BOC flow meter (A) and a pressure controller. The gas flow facilitated spore detachment from conidiophores and carried them toward the outlet, where plasma‐modified filters (AAC, POX, OD, and PFO) and non‐coated controls (NC) were positioned in a filter holder to capture airborne spores.

Exhaust gas was vented through a bubbling bottle containing 80% ethanol to minimize contamination, with a second BOC flow meter (B) monitoring outlet flow to ensure consistency. Each run was conducted for 30 min, after which filters were aseptically removed and transferred to six‐well plates for microscopy. Negative controls were performed without fungal colonies. All experiments were conducted in triplicate, including three chemical, three technical, and three biological replicates.

### 5.6 Microscopic Analysis

#### 5.6.1 Inverted Microscopy

Following the spore capture experiment in the custom‐made aerosolization chamber, the filters were observed via microscopy to visualize the captured fungal spores. Different fungal species, *A. niger*, *Cladosporium* sp., *P. roqueforti*, and *R. glutinis* were sampled. The NC, [AAC, POX, OD, PFO (16 and 50‐nm coated)] filters were subsequently analyzed using an inverted microscope.

A Nikon ECLIPSE T*i*2 Inverted research microscope was used to observe the fungal spores captured on the modified filters, providing high‐resolution imaging. 4x, 10x, and 40x objectives were used to provide total magnification of 40x, 100x and 400x. The images were taken before and after sampling with fungal spores. The non‐coated and control filters were also imaged during the experiments.

#### 5.6.2 Scanning Electron Microscopy

An Inspect F50 Scanning electron microscope (SEM) was used to further characterize the fungal spores captured by the filters. The SEM is equipped with a field‐emission gun electron emitter, which is used to acquire secondary‐electron images at an accelerating voltage of 2 kV and a working distance of 10 mm.

### 5.7 Quantification Methods

Two approaches were used to quantify fungal spores captured on plasma‐coated and non‐coated filters. Filters were pre‐weighed in 15 mL centrifuge tubes prior to sampling and reweighed post‐sampling to determine mass changes. Each filter was then immersed in 4 mL of 1X phosphate‐buffered saline (PBS) to ensure complete submersion and vortexed at 2500 rpm for 5 min to detach the captured spores. 1X PBS was prepared from a 20X stock by diluting it 1:20, mixing 50 mL of 20X PBS with 950 mL of deionized water to obtain 1 L of 1X PBS. The resulting PBS eluate was used for subsequent spore enumeration and weight analysis. After washing, the filters were dried overnight and examined under a microscope to confirm complete removal of spores.

#### 5.7.1 Spore Count by Hemocytometer

Spore counting was performed using a hemocytometer following standard procedures [70]. Briefly, 10 µL of the above‐mentioned spore suspension was added to the reference grids of a hemocytometer slide on both sides. The full grid on a hemocytometer contains nine squares, each of which has an area of 1 mm and a depth of 0.1 mm, resulting in a volume of 0.0001 mL (0.1 mmm^3^) per large square. The central counting area of the hemocytometer contains 25 large squares. The number of spores present in a total of 5 large squares on both sides of the hemocytometer was counted and used to calculate the average number of spores present in one large square. The spore concentration was calculated by using the formula given below:

$$\begin{array}{cc} \mathrm{Spore}\;\mathrm{Concentration}\;\left(\frac{\mathrm{spores}}{\mathrm{mL}}\right) & =\frac{\mathrm{Total}\;\mathrm{number}\;\mathrm{of}\;\mathrm{spores}\;\mathrm{counted}}{\mathrm{Number}\;\mathrm{of}\;\mathrm{squares}\;\mathrm{counted}}\\ & \;\times \frac{\mathrm{Dilution}\;\mathrm{factor}\;}{\mathrm{Volume}\;\mathrm{of}\;\;a\;\mathrm{single}\;\mathrm{square}\;\;\left(\mathrm{in}\;\mathrm{mL}\right)} \end{array}$$

#### 5.7.2 Dried Spore Pellet Weight

The remaining spore suspension was transferred to pre‐weighed sterile 15 mL tubes and centrifuged at 4500 rpm for 5 min to pellet the spores. The supernatant was discarded, and the pellet was washed twice with 1 mL sterile deionized water to ensure sample purity. Tubes were then dried overnight in a biosafety cabinet to remove residual moisture. Dried samples were weighed using an analytical balance (precision ± 0.1 mg), and spore mass was determined by subtracting the initial tube weight from the final weight. All measurements were performed in triplicate, and mean values with standard deviations were calculated. Blank filter controls (processed without fungal spores) were included to account for background residues such as filter fibers. Dried spore samples were stored at −20°C for subsequent molecular analyses.

### 5.8 Filter Efficiency and Spore Loss Test

An Andersen cascade impactor was used to evaluate the performance of the custom air sampling system and to determine the spore capture efficiency of coated and non‐coated PET filters. The impactor was placed inside a Class II biosafety cabinet and connected to an air pump operating at a constant flow rate of 28.3 L min^−^^1^ for 3 min per sample. The inlet was connected via tubing to the custom aerosolization chamber (Figure S12) containing the fungal colony, while the outlet was linked to a bubbling system to minimize contamination. The impactor's multiple stages, each containing agar plates, allowed for size‐selective deposition of airborne spores. To evaluate spore loss for the EPM2000 membranes, sampling was performed for 30 min at a constant flow rate of 17.8 L min^−^^1^ under a pressure of 1.5 bar. The outlet of the sampling chamber was connected to an Andersen cascade impactor equipped with agar plates. After each sampling, plates were aseptically removed and incubated for 2–3 days before colony counting. Initial tests were performed without filters to establish a control for spore recovery. Subsequent experiments incorporated non‐coated (NC) and plasma‐coated (POX and PFO) filters/membranes between the colony and the impactor. All experiments were performed in triplicate for each fungal species across three independent days to ensure reproducibility.

### 5.9 Different Filter Geometry

Commercially available Whatman EPM2000 air sampling filters were plasma‐coated as per the conditions described in Table 2. These filters were subsequently used to sample airborne fungal spores, as described above. This final test allows for a comparative analysis of different filter types. The EPM2000 filters are made of 100% pure borosilicate glass; they have a defined pore size of 0.3 µm, and their shape is flat.

### 5.10 Statistical Analysis

Overall data were analyzed using GraphPad Prism software 10.4.0 (621). Two‐way ANOVA was used to evaluate the significance of the data obtained. An uncorrected Fisher's LSD test was applied to compare spore concentrations between the NC (control) and each plasma coating (AAC, POX, OD, and PFO) for all four fungal species. In all cases, *p*‐values of ≤0.05 were used for a significant difference.

## Author Contributions

All authors contributed to the study conception and design. Material preparation, data collection, and analysis were performed by Hafiza Umaima Affan. The first draft of the manuscript was written by Hafiza Umaima Affan, and all authors commented on previous versions of the manuscript. All authors read and approved the final manuscript.

## Funding

This work was supported by the Australian Research Council Future Fellowship Grant (FT200100301).

## Conflicts of Interest

The authors declare no conflict of interest.

## Supporting information




**Supporting File**: gch270127‐sup‐0001‐SuppMat.pdf.

## Data Availability

The data that support the findings of this study are available from the corresponding author upon reasonable request.
