# Supplementary material for: Surface‐Engineered Filters for Wettability‐Driven Collection of Airborne Fungal Spores
Source: Glob Chall. 2026 Jul 1;10(7):e70127. doi: 10.1002/gch2.70127 (PMC13323565; doi:10.1002/gch2.70127)
Supplement: Supplementary file 1 — Supporting File: gch270127‐sup‐0001‐SuppMat.pdf. [file GCH2-10-e70127-s001.pdf]

Surface-Engineered Filters for Wettability-driven Collection of Airborne Fungal Spores

Authors:

Hafiza Umaima Affan, * [a, b] Claire Lenehan,[a, b] Sally Fryar, [a] Michael Taylor, [a, d,] Harriet Whiley, [a] Iliana Delcheva, [a,b] Melanie MacGregor * [a, b, c]

Affiliations:

[a] College of Science and Engineering, Flinders University, Sturt Road, Bedford Park, South Australia, 5042, Australia

[b] Flinders Institute for Nanoscale Science and Technology, Flinders University, Sturt Road, Bedford Park, South Australia, 5042, Australia

[c] Nano and Microplastics Research Consortium, Flinders University, Sturt Road, Bedford Park, South Australia, 5042, Australia

[d] WSP Australia Pty Limited, Adelaide, South Australia, 5000, Australia

* Corresponding Authors

melanie.macgregor@flinders.edu.au

hafizaumaima.affan@flinders.edu.au

Supplementary Figures

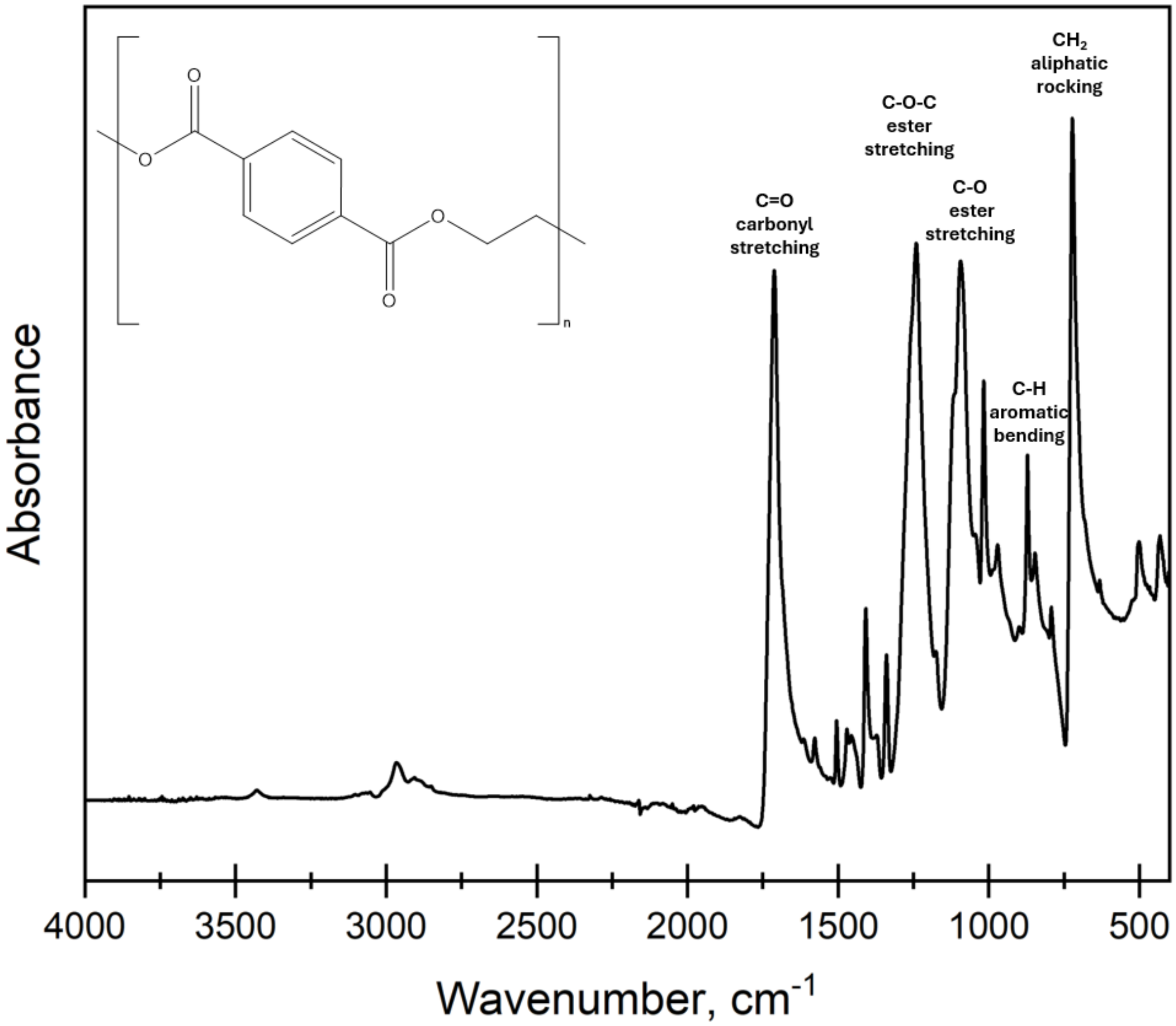

Figure S1 FTIR-ATR spectrum of the filter material with the characteristic peaks associated with PET labelled. Chemical structure of PET provided in the top left corner.

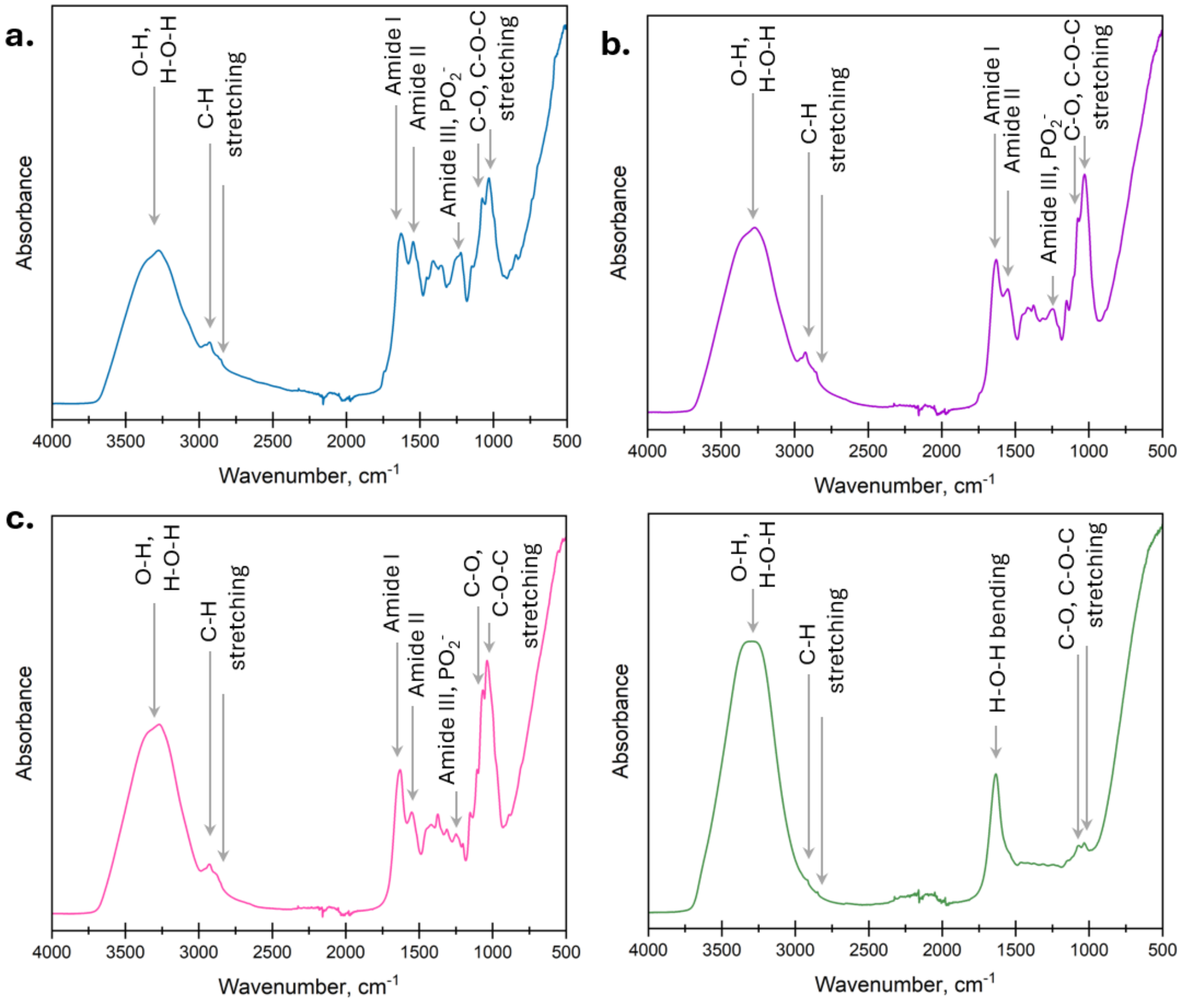

Figure S2 ATR-FTIR spectrum collected on spores of *Aspergillus niger* (a), *Cladosporium* sp. (b), *Penicillium roqueforti* (c), and *Rhodotorula glutinis* (d)

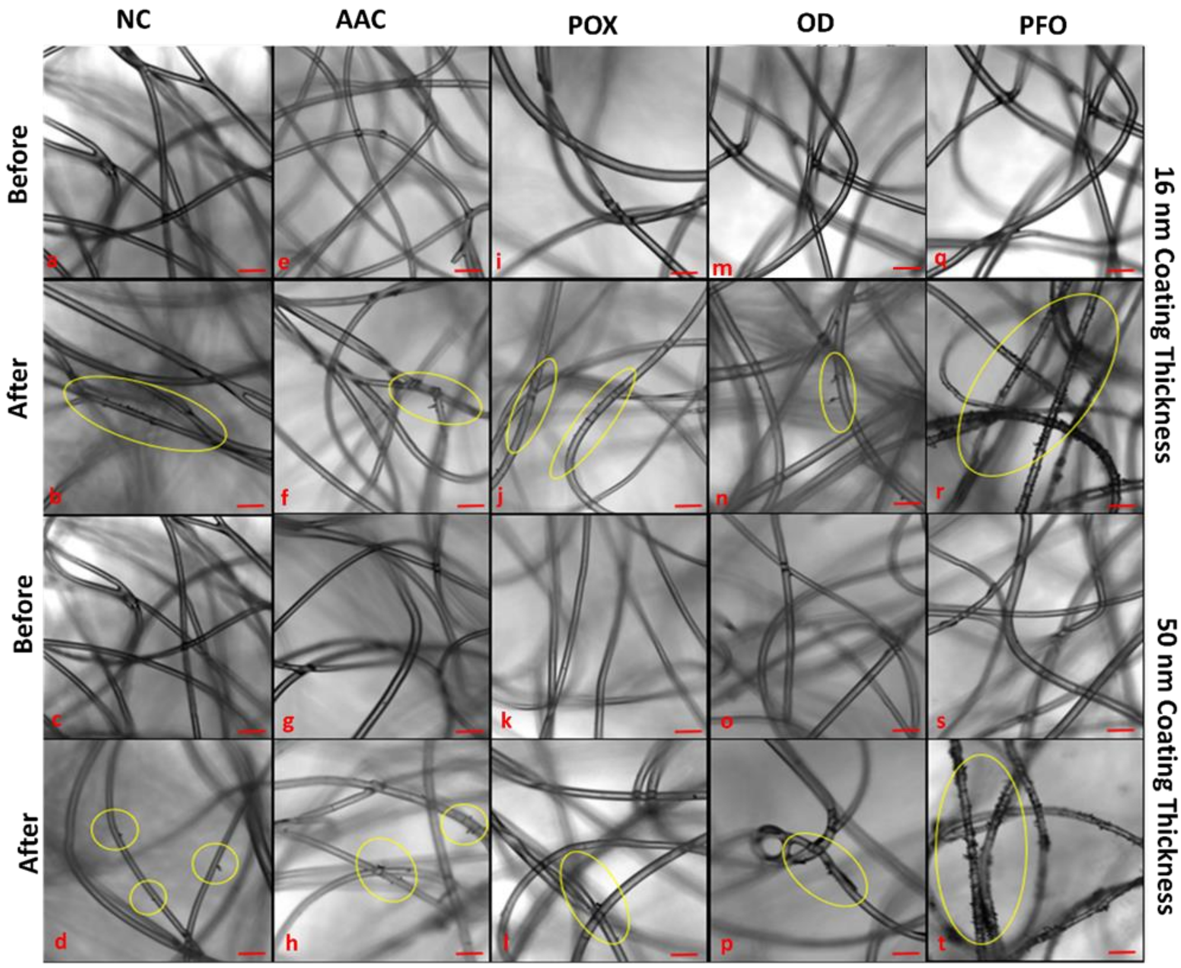

Figure S3 Microscopic images of *Aspergillus niger* spores captured on 16 nm and 50 nm coated and noncoated filters at 100x magnification. (a-d) non-coated filters (e-h) AAC coated filters (i-l) POX coated filters (m-p) OD coated filters (q-t) PFO coated filters. Rows representing before and after images. Columns representing different coatings (NC, AAC, POX, OD, and PFO). The scale bar was 200 μm.

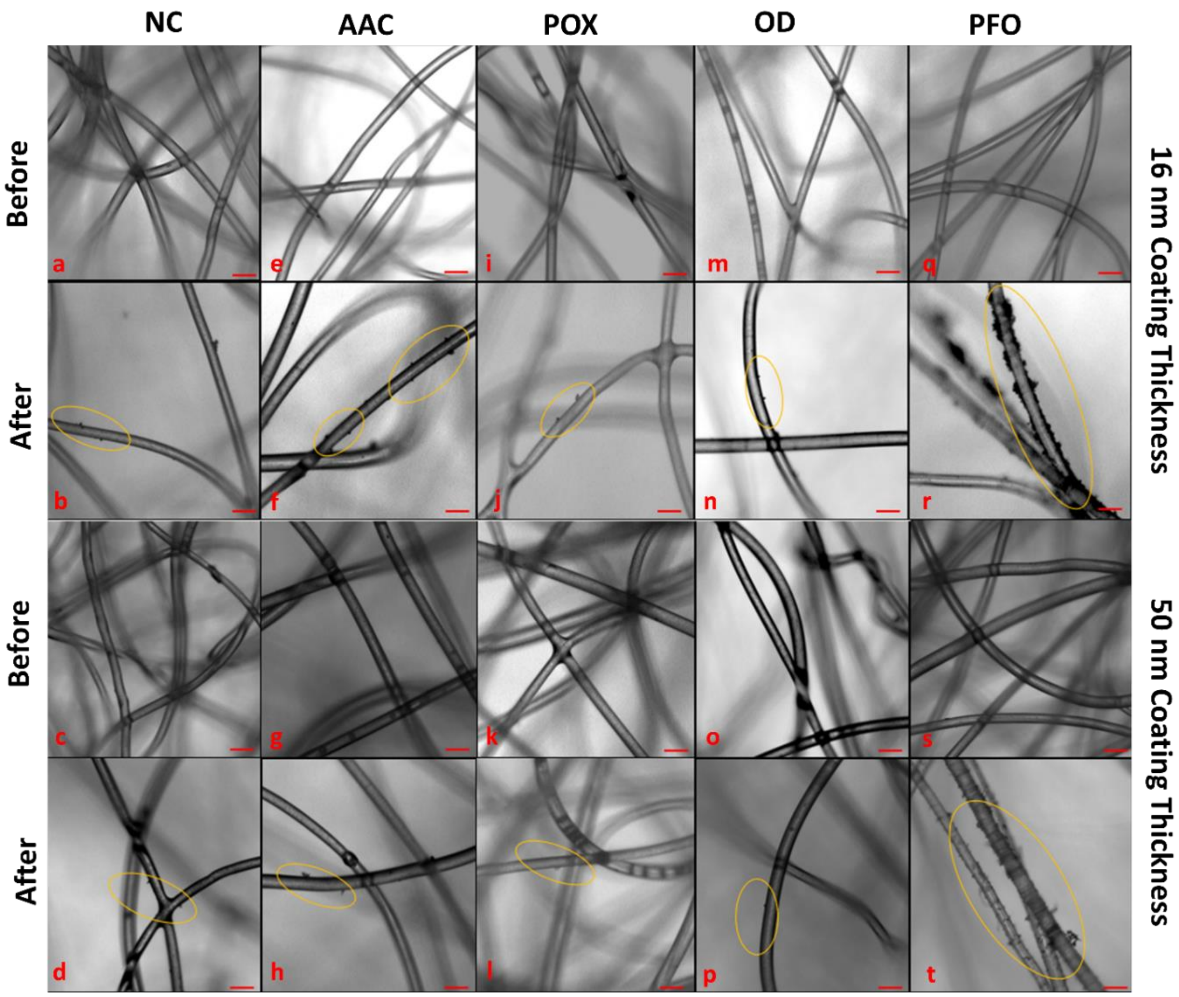

Figure S4 Microscopic images of *Cladosporium* sp., spores captured on 16 nm and 50 nm coated and noncoated filters at 100x magnification. (a-d) non-coated filters (e-h) AAC coated filters (i-l) POX coated filters (m-p) OD coated filters (q-t) PFO coated filters. Rows representing before and after images. Columns representing different coatings (NC, AAC, POX, OD, and PFO). The scale bar was 200 μm.

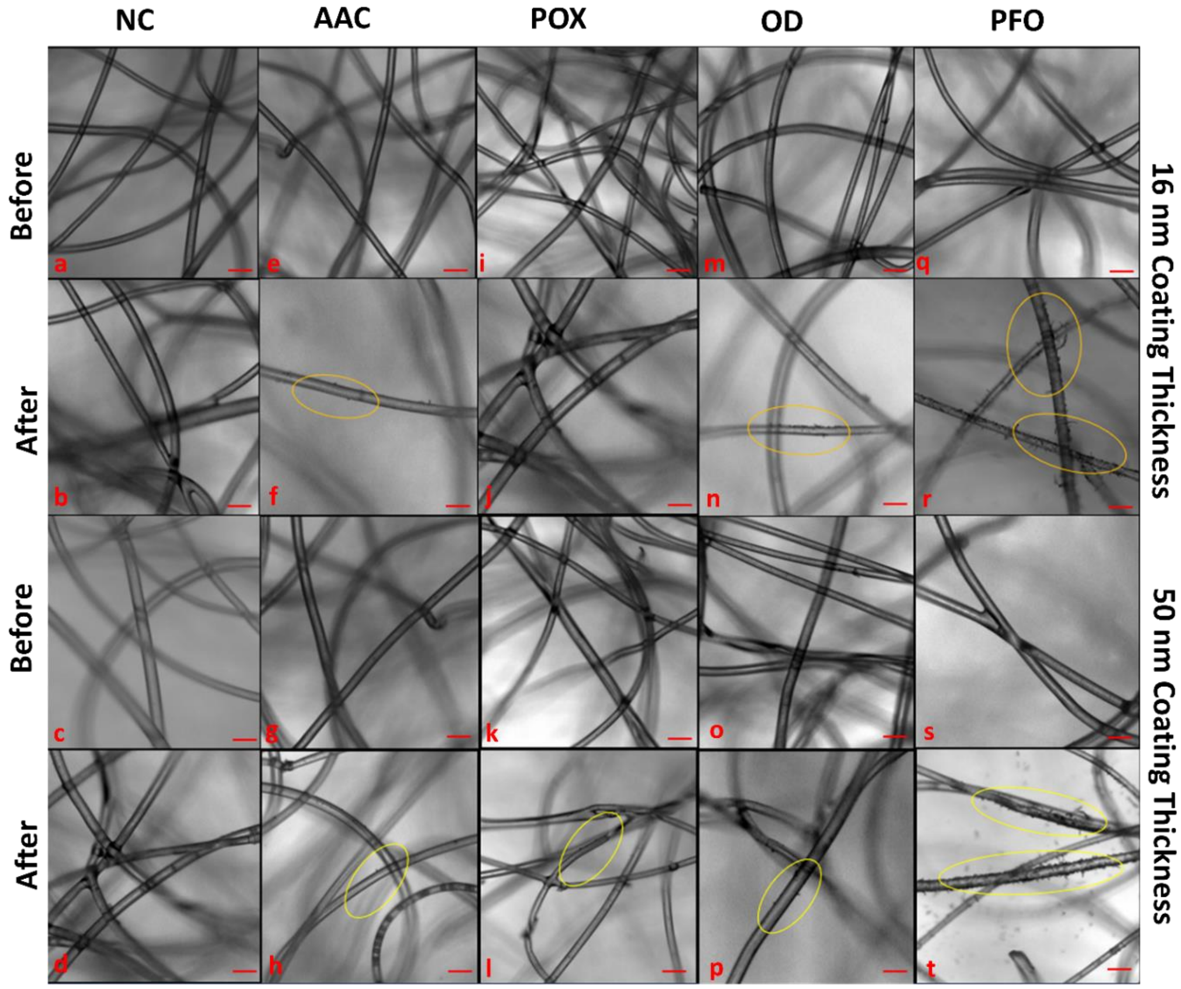

Figure S5 Microscopic images of *Penicillium roqueforti* spores captured on 16 nm and 50 nm coated and noncoated filters at 100x magnification. (a-d) non-coated filters (e-h) AAC coated filters (i-l) POX coated filters (m-p) OD coated filters (q-t) PFO coated filters. Rows representing before and after images. Columns representing different coatings (NC, AAC, POX, OD, and PFO). The scale bar was 200 μm.

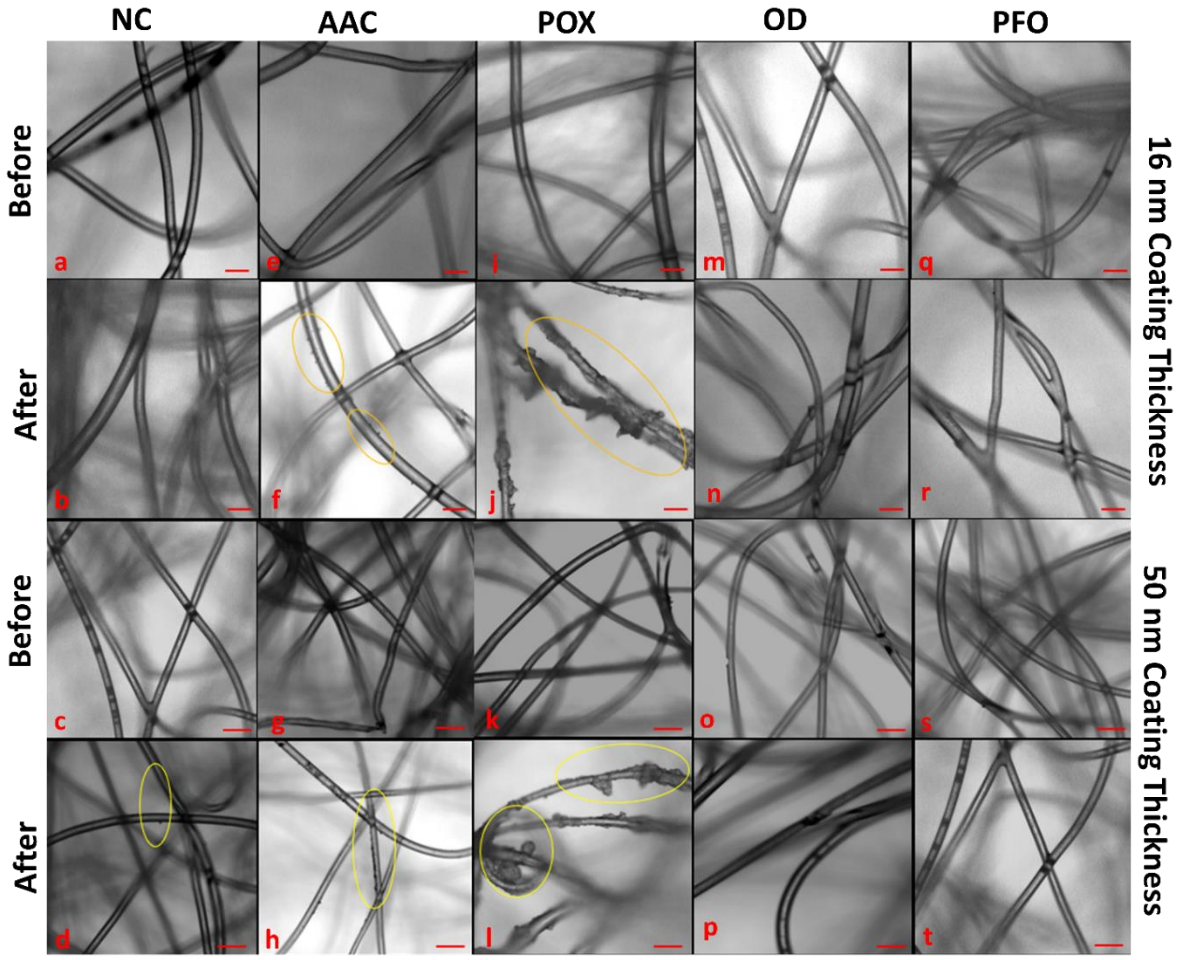

Figure S6 Microscopic images of *Rhodotorula glutinis* spores captured on 16 nm and 50 nm coated and noncoated filters at 100x magnification. (a-d) non-coated filters (e-h) AAC coated filters (i-l) POX coated filters (m-p) OD coated filters (q-t) PFO coated filters. Rows representing before and after images. Columns representing different coatings (NC, AAC, POX, OD, and PFO). The scale bar was 200 μm.

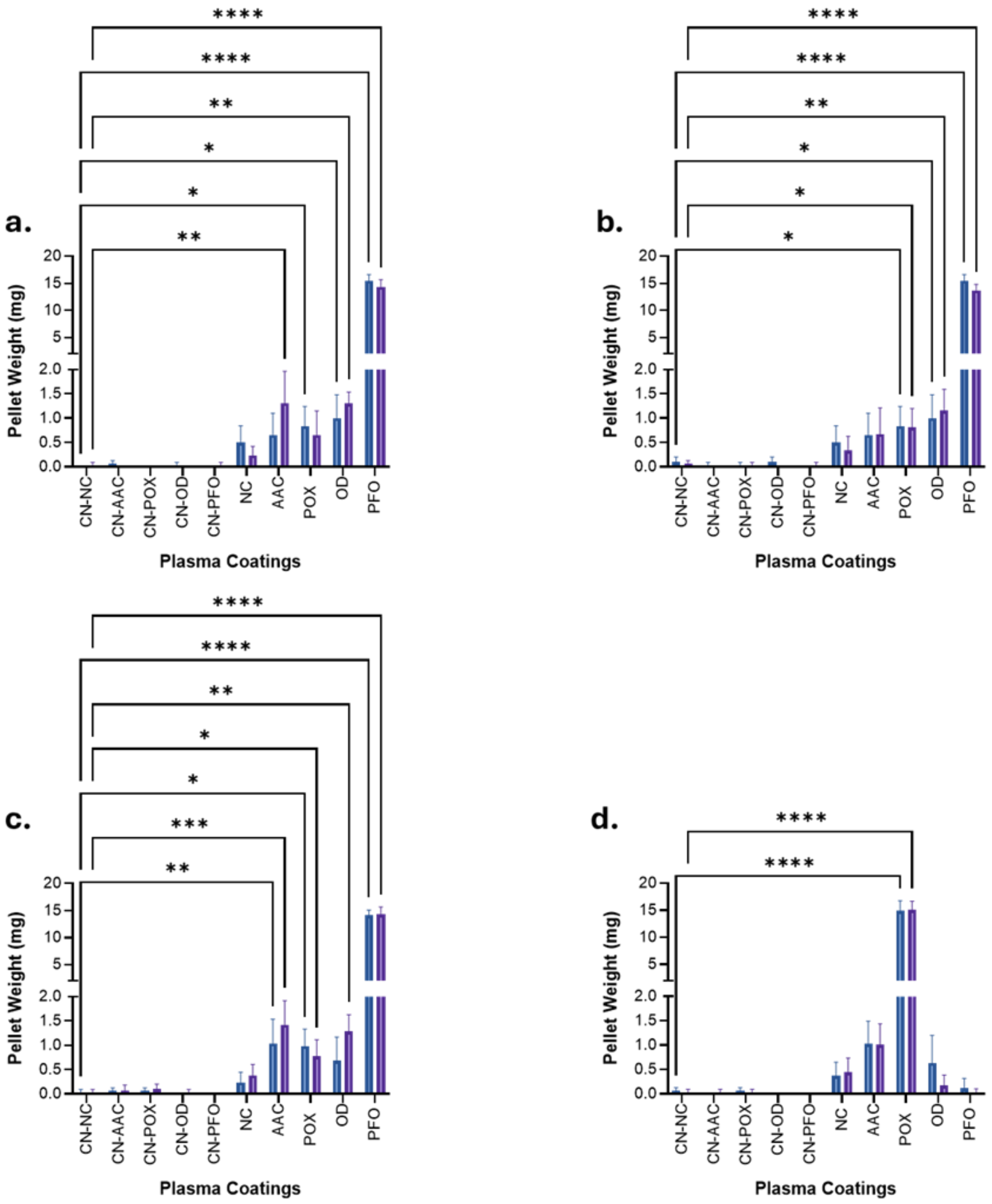

Figure S7 Comparison of control filters (no spores) and filters exposed to fungal spores. Control filters (CN, no spores) are shown for both non-coated and coated filters, alongside corresponding filters sampled with fungal spores. Panels show results for: (a) *Aspergillus niger* (b) *Cladosporium* sp. (c) *Penicillium roqueforti* (d) *Rhodotorula glutinis*. Significance was determined via two-way ANOVA with multiple comparisons Uncorrected Fisher's least-significant difference test. P-value = * ≤0.05, ** ≤0.01, **** ≤0.0001

Table S1 Recovery efficiency (%) of the PBS vortex elution step for fungal biomass collected on coated and uncoated PET filters. Values are mean percentage recovery ± average deviation of the mean, *n* = 9. Green values indicate high-biomass conditions where recovery was assessed well above the balance precision (0.1 mg). Greater variability was observed when very small amounts of biomass were captured, particularly below approximately 0.5 mg, where small absolute weighing differences become amplified in the percentage recovery calculation. Under these low-mass conditions, the median recovery remained close to 100%, and apparent outliers are therefore attributed primarily to the limited precision of the mass measurement rather than inefficient vortex elution.

Specie	NC	AAC	POX	OD	PFO
16 nm coating					
A.Niger	60±28	80±19	85±13	86±11	**99±1**
cladosporium sp.	98±3	75±33	94±8	99±1	**100**
P. Roqueforti	67±29	90±11	93±10	81±20	**99±1**
R. Glutinis	97±5	98±5	**99±1**	93±11	95±6
50 nm coating					
A.Niger	44±28	78±12	87±15	92±8	**99±2**
cladosporium sp.	96±6	90±14	100	97±4	**99±1**
P. Roqueforti	84±18	94±7	84±16	90±10	**98±1**
R. Glutinis	88±17	95±8	**100**	73±32	100

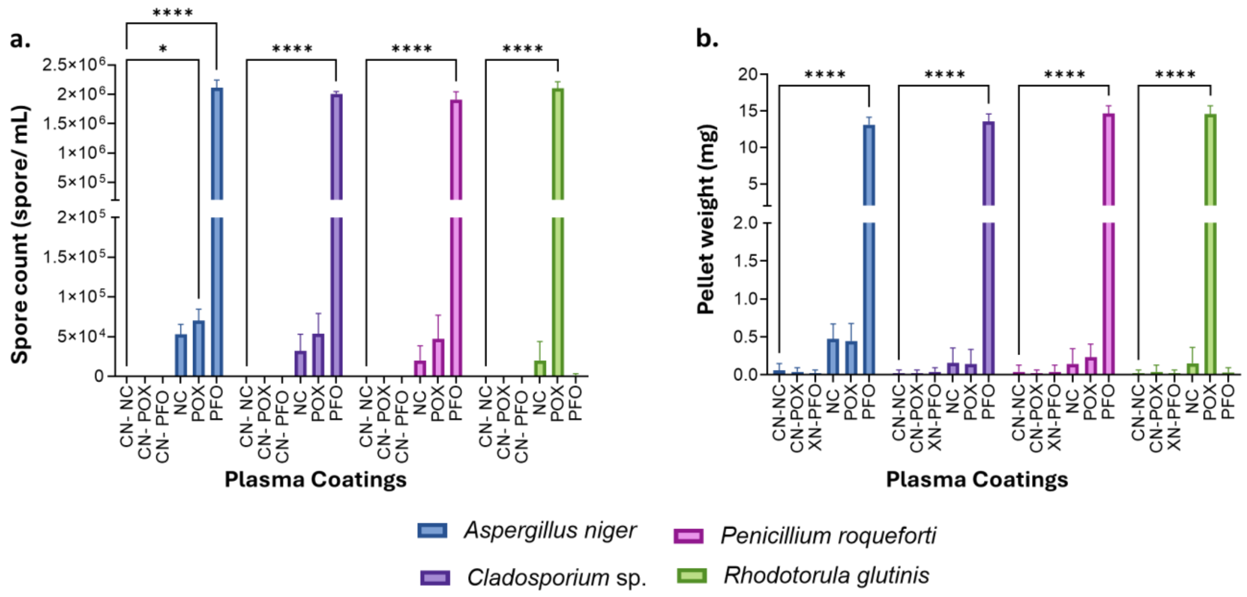

Figure S8 (a) Concentration of 4 fungal species spores extracted from 16 nm coated and non-coated EPM2000 membranes. (b) Dry pellet weight of different fungal species spores extracted from EPM2000 membranes (controls & treated filters). Significance was determined via two-way ANOVA with multiple comparisons Uncorrected Fisher's least-significant difference test. P-value = * ≤0.05, ** ≤0.01, **** ≤0.0001.

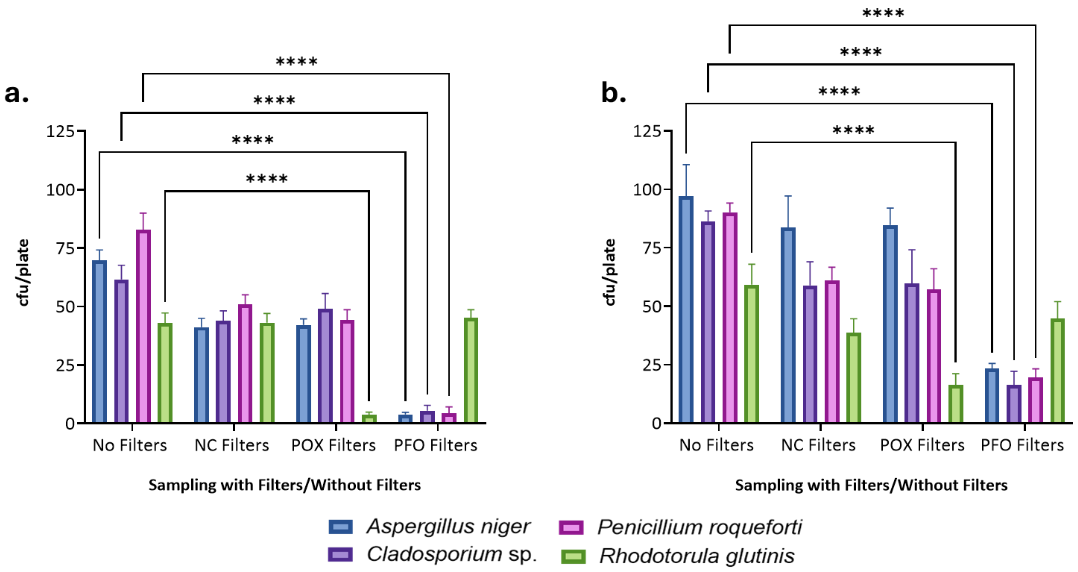

Figure S9 Positive-hole correction graphs to adjust colony counts from 400-hole impactor for the possibility of collecting multiple particles through a hole. (a) PET filters (b) EPM2000 membranes. Significant differences in capture efficiency were observed among filter types. Data represent mean ± SD. Significance was determined via two-way ANOVA with multiple comparisons Uncorrected Fisher's least-significant difference test. P-value = * ≤0.05, ** ≤0.01, **** ≤0.0001.

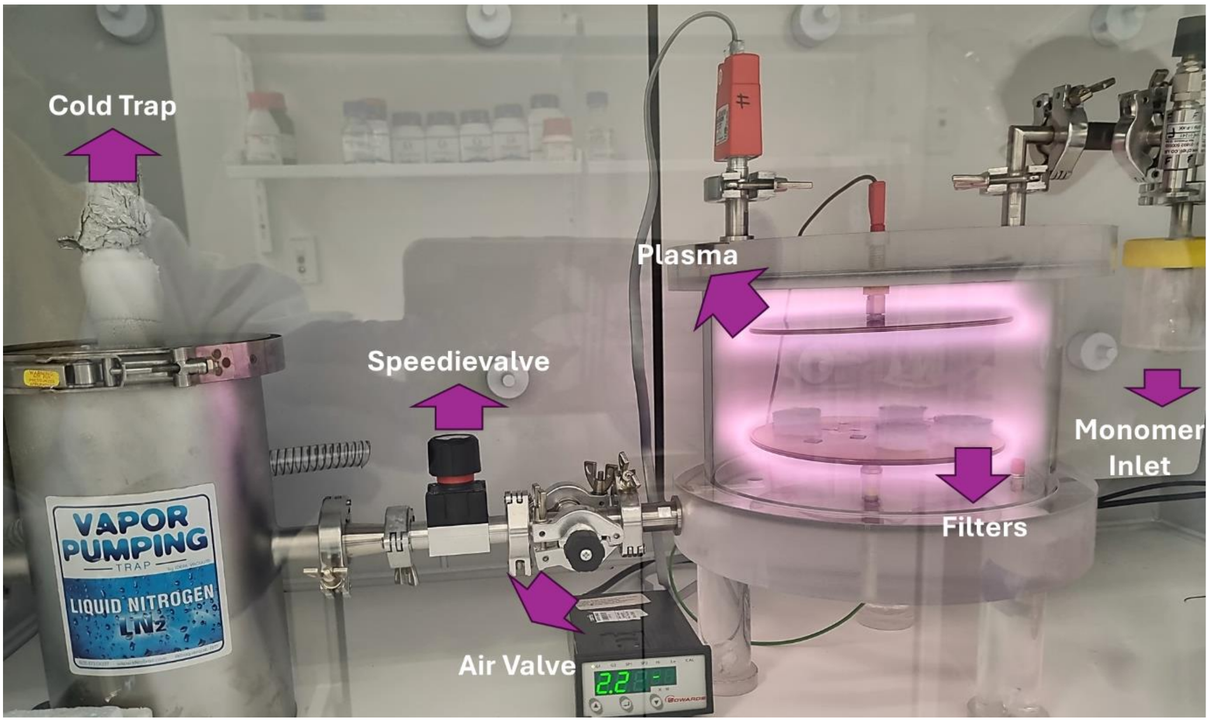

Figure S10 Custom-made plasma reactor used for the deposition of nanothin coatings from AAC, POX, OD, and PFO onto the filters surface.

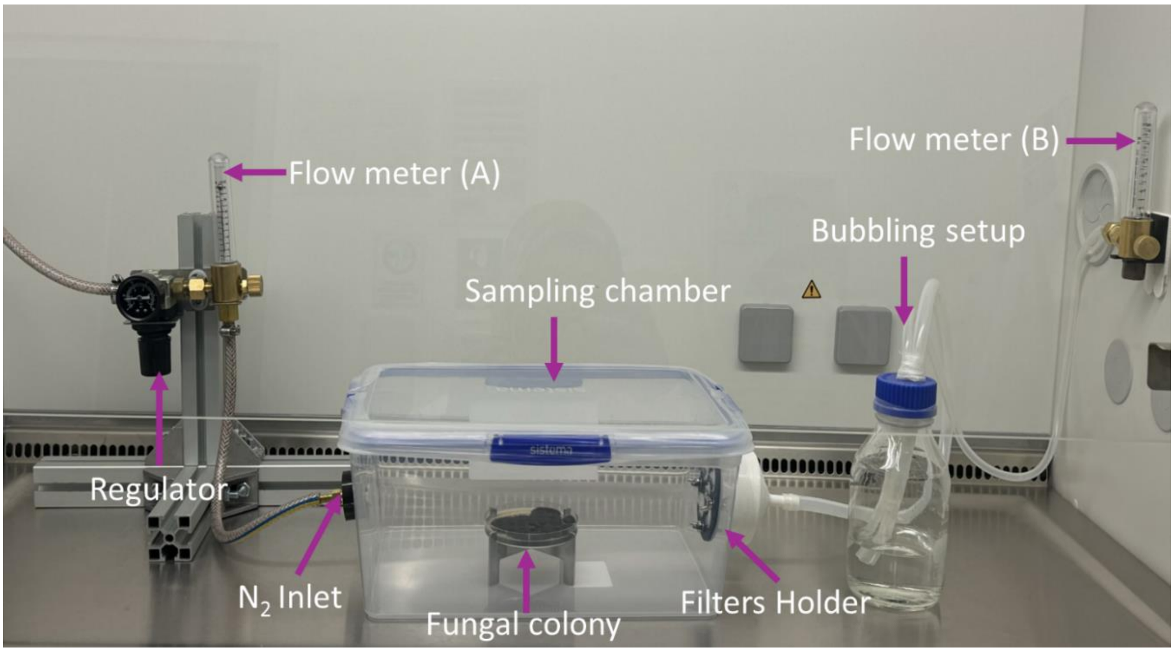

Figure S11 Custom-made experimental setup to capture aerosolized fungal spores of different fungi species. The setup was placed inside the biosafety cabinet.

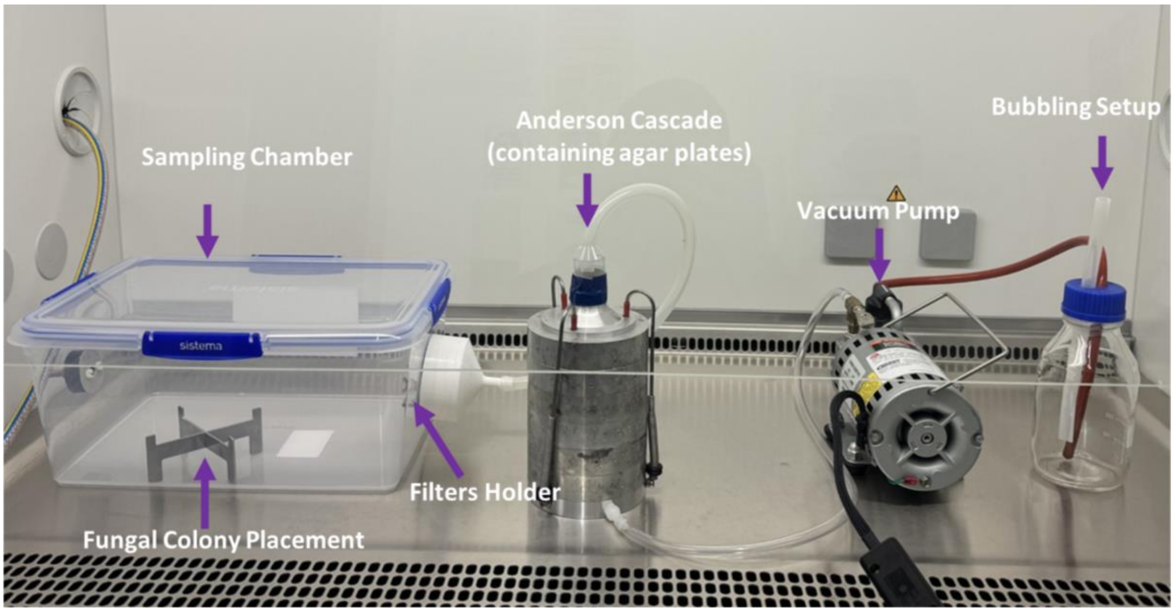

Figure S12 Andersen cascade impactor setup to evaluate the performance of the custom air sampling system and to determine spore capture efficiency and spore loss of filters and membranes.
